# Antimicrobial drug use in food-producing animals and associated human health risks: what, and how strong, is the evidence?

**DOI:** 10.1186/s12917-017-1131-3

**Published:** 2017-07-04

**Authors:** Karin Hoelzer, Nora Wong, Joe Thomas, Kathy Talkington, Elizabeth Jungman, Allan Coukell

**Affiliations:** The Pew Charitable Trusts, 901 E Street NW, Washington, DC 20004 USA

**Keywords:** Antimicrobial drug use, Farm animals, Antimicrobial resistance, Public health risk

## Abstract

**Background:**

Antimicrobial resistance is a public health threat. Because antimicrobial consumption in food-producing animals contributes to the problem, policies restricting the inappropriate or unnecessary agricultural use of antimicrobial drugs are important. However, this link between agricultural antibiotic use and antibiotic resistance has remained contested by some, with potentially disruptive effects on efforts to move towards the judicious or prudent use of these drugs.

**Main text:**

The goal of this review is to systematically evaluate the types of evidence available for each step in the causal pathway from antimicrobial use on farms to human public health risk, and to evaluate the strength of evidence within a ‘Grades of Recommendations Assessment, Development and Evaluation‘(GRADE) framework. The review clearly demonstrates that there is compelling scientific evidence available to support each step in the causal pathway, from antimicrobial use on farms to a public health burden caused by infections with resistant pathogens. Importantly, the pathogen, antimicrobial drug and treatment regimen, and general setting (e.g., feed type) can have significant impacts on how quickly resistance emerges or spreads, for how long resistance may persist after antimicrobial exposures cease, and what public health impacts may be associated with antimicrobial use on farms. Therefore an exact quantification of the public health burden attributable to antimicrobial drug use in animal agriculture compared to other sources remains challenging.

**Conclusions:**

Even though more research is needed to close existing data gaps, obtain a better understanding of how antimicrobial drugs are actually used on farms or feedlots, and quantify the risk associated with antimicrobial use in animal agriculture, these findings reinforce the need to act now and restrict antibiotic use in animal agriculture to those instances necessary to ensure the health and well-being of the animals.

## Background

### Antimicrobial use and resistance in animal agriculture

Antimicrobial resistance is a global public health threat, reflected in at least 2 million resistant infections and at least 23,000 deaths in the United States each year [1]. Antimicrobial drug use is considered to be the single most important factor leading to resistance [1]. However, effective policy interventions are hindered by the fact that the emergence and spread of antimicrobial resistance is complex and the underlying dynamics incompletely understood [2, 3]. Antimicrobial drugs are used in a variety of settings including hospitals, outpatient clinics, and long-term care facilities as well as animal-associated settings such as veterinary clinics, farms, and feedlots. There is general scientific consensus that antimicrobial drug use in a variety of settings contributes to the burden of antimicrobial resistance [2, 3]. However, the relative contribution of different antimicrobial uses to the overall burden has so far remained unclear. Moreover, despite decades of research and a considerable body of scientific evidence, the link between antimicrobial drug use on farms and antimicrobial resistant infections in humans remains contested by critics, primarily in the U.S. [3]. This dispute is disruptive to the debate around judicious use of antimicrobial drugs in animal agriculture, and has the potential to slow the implementation of policies aimed at assuring the responsible and prudent use of antibiotics in animal agriculture. Notably, various organizations including the World Health Organization for Animals (OIE), U.S. Food and Drug Administration, and the British and American Veterinary Medical Associations, have defined the concept of responsible, prudent or judicious antimicrobial use in animal agriculture. Optimizing therapeutic efficacy and minimizing antimicrobial resistance are key objectives underpinning all of these definitions, despite some definitional differences. For the purposes of this study, judicious antimicrobial use shall be equivalent to OIE’s ‘responsible and prudent use’ definition of ‘improv[ing]animal health and animal welfare while preventing or reducing the selection, emergence and spread of antimicrobial-resistant bacteria in animals and humans.’

### Policy solutions to ensure judicious antimicrobial use in animal agriculture

Around the world, policy efforts to ensure judicious antimicrobial use in animal agriculture have generally, as a first step, focused on restricting the use of those antimicrobial drugs important for human medicine for ‘growth promotion’ purposes. For the purposes of this article, the Codex Alimentarius definition of growth promotion shall be used, where ‘growth promotion refers to the use of antimicrobial substances to increase the rate of weight gain and/or the efficiency of feed utilization in animals by other than purely nutritional means’ and antimicrobials are thus administered to healthy animals with the primary goal of increasing growth rates and enhancing feed conversion efficiency [4].

As early as 1969, a report by the U.K. Joint Committee on the Use of Antibiotics in Animal Husbandry, known as the ‘Swann Report’, motivated by concerns about the emergence of antimicrobial resistance, called for the ban of medically important (i.e., shared between humans and animals) antimicrobials for growth promotion purposes [3]. However, it took nearly another two decades before governments took concrete action and individual countries differ drastically in their responsiveness on the issue [3]. Sweden outlawed the use of all antimicrobial drugs for growth promotion in 1986 and Denmark banned the use of the two medically important antimicrobials, avoparcin and virginiamycin, as growth promoters in 1995 [5]. Avoparcin was banned for growth promoting purposes across the European Union in 1997 and growth promotion uses of the remaining four medically important antibiotics (as defined by the World Health Organization [6]) bacitracin, spiramycin, tylosin and virginiamycin were banned in 1999 [5]. Following the precautionary principle, the use of any antimicrobial drug for growth promotion, including antimicrobial drug classes not used in human medicine, has been banned in Europe since 2006 [7]. In the U.S., the use of medically important antimicrobial drugs (as defined by the U.S. Food and Drug Administration [8]) for growth promotion was phased out on January 1, 2017. In fact, according to data collected by the OIE for 2015, more than 70% of member countries that responded to the survey did not authorize antimicrobial drugs for growth promotion purposes [9]. However, outlawing the use of medically important antimicrobial drugs for growth promotion is only the first step towards assuring their judicious use in animal agriculture. Several European countries have taken concrete next steps towards curtailing the emergence and spread of antimicrobial resistance by assuring antimicrobial drugs are used judiciously and only when necessary to ensure the health and well-being of the animal, and the U.S. Food and Drug Administration has recently announced plans to examine potential actions to that effect [10].

### The dynamics of antimicrobial resistance and their relevance for this study

For the purpose of this study, antimicrobial resistance is defined as ‘microbiological resistance’ and refers to the increased resistance of a bacterium in vitro compared to a population of wild-type bacteria which can be expressed, for instance, as an increase in minimal inhibitory concentration (MIC). Infections with bacteria that meet this definition of resistance may in fact on occasion still respond to treatments with the antimicrobial drug. In this respect, microbiological resistance is different from the concept of ‘clinical resistance’, which is focused on treatment failures and considers clinical factors such as the therapeutic concentration that can be reached at the site of infection. To evaluate clinical resistance, MIC values can be compared to clinical breakpoints, which should be specific to the animal species and site of infection [11]. However, for many situations encountered in veterinary medicine specific breakpoints have not been established and extrapolations from existing breakpoints can be challenging.

This study focuses on the emergence and spread of acquired antimicrobial resistance. This resistance can emerge through point mutations, usually associated with a progression from low-level to high-level resistance as sequential mutations occur [12], or through horizontal gene transfer (HGT), which usually shows immediate high-level resistance as resistance genes are shared among bacteria [12]. HGT can occur through several mechanisms [12]; for instance, plasmids that carry resistance genes can be shared among bacterial strains, bacteriophages can transfer resistance genes from one bacterium to another, and bacteria can take up naked DNA (e.g., genes originating from dead bacteria). Notably, the relative importance of each resistance pathway appears to at least partially depend on the bacterial species [12]. Acquired resistance has to be distinguished from inherent resistance. Some bacteria are inherently resistant to a drug because the bacterium is outside of the drug’s spectrum of action, for example because the bacterium lacks the drug target. In addition, some bacteria can be transiently resistant to a drug without a corresponding genetic change, probably because of a transient dormant state in which the bacterium’s metabolism is lowered to a point where it virtually ceases to function. These resistance types are generally believed not to be affected by antimicrobial use and will not be considered here.

The analysis of the link between antibiotic use on farms and human health risk is complicated by the fact that the evolutionary dynamics of antimicrobial resistance do not followed a simple ‘necessary and sufficient’ [13] model of epidemiological causation, where the presence of antimicrobial drug use would be both necessary and sufficient for the emergence of resistance. In many cases, the same acquired antimicrobial resistance type or trait can be encoded by more than one genetic mechanism. Resistance traits often, although not always, carry a fitness cost, at least in in vitro experimental systems, and fitness costs may differ for the same resistance trait based on the determining genetic changes [12]. If there is a fitness cost associated with a resistance trait, resistant strains are selected *against* in antimicrobial-free environments and selected *for* in the presence of antimicrobials. The fitness cost may be higher for chromosomally-mediated than plasmid-mediated mutations, may differ by resistance mechanism (e.g., whether resistance is conferred by modification of a metabolic pathway, alteration of a target site, or upregulation of membrane channels), and may increase with the number of point mutations required to express the trait [12]. In some cases, however, the fitness cost is very low. Moreover, compensatory mutations that correct the fitness loss brought about by the resistance-conferring mutations are possible. It is unknown how closely fitness costs under in vitro conditions track those experienced in vivo – for instance, multi-drug resistance may be associated with a lower fitness cost in vivo than predicted based on in vitro data [14].

Antimicrobial resistance is not a new phenomenon. There is strong scientific evidence for the pre-existence of some antimicrobial resistance genes (e.g., beta-lactamase genes) in the absence of any antimicrobial use [14]. In fact, many antimicrobial drugs are naturally produced by fungi or bacteria to stave off competition from other bacteria; some resistance genes are necessary to allow these antimicrobial-producing microbes to survive, and others have emerged as an adaptation to the natural presence of these antimicrobial compounds in the environment, primarily in soil [15]. The term ‘resistome’ has been proposed to describe the ecology of resistance genes, broadly encompassing all resistance genes circulating in pathogenic and non-pathogenic bacteria, be they from soil, animals, humans, or other sources [15]. A comprehensive knowledge of the resistome and adequate phylogenetic analysis is necessary to determine whether a newly-detected resistance gene truly emerged recently, has been present for a while but recently became more prevalent, or simply has not been detected previously.

The evolutionary dynamics of antimicrobial resistance are further complicated by the potential for cross-resistance (the simultaneous resistance to multiple related drugs that share a drug target) and co-resistance (where several genes are transferred together, for instance on one plasmid, and selection for one of the genes indirectly selects for the others as well). Moreover, antimicrobial resistance to a given drug can be mediated by multiple genetic changes, with potentially different evolutionary dynamics, and bacterial mutation rates vary, potentially pre-disposing some bacteria (i.e., ‘hypermutators’) to the more rapid emergence of resistance-conferring mutations than others.

For all of these reasons, the exact dynamics between antimicrobial use and resistance development may differ by bacterial species, drug or drug target, and resistance-conferring mutation, and may be influenced by external factors such as the selection pressures exerted by the use of related drugs. Even if antimicrobial use decreases or ceases, this may not necessarily translate into a direct, measurable drop in antimicrobial resistance, and the impact of introducing, restraining or eliminating antimicrobial use may vary by bacterial strain, bacterial target and environment (e.g., as a result of different resistance mechanisms with different evolutionary and ecological properties). Perhaps most importantly, resistance may not in all cases be reversible by discontinuing antimicrobial use alone. As is also clear from this discussion, there are many aspects about the emergence, ecology and evolution of antimicrobial resistance genes that are not yet fully understood. For these reasons it is difficult to extrapolate from individual research studies to other settings, and to adequately evaluate all direct and indirect impacts of antimicrobial use on the emergence of resistance.

Because antimicrobial drug use and the emergence of resistance may not follow a direct cause and effect relationship, it can be very difficult to establish causality outside of tightly controlled experimental settings. However, many chronic diseases also do not follow a direct cause and effect relationship and research methods developed to address these challenges may be applicable to antimicrobial resistance. One widely used epidemiological model of causation that fits these chronic diseases and antimicrobial resistance is that of ‘sufficient-component causes,’ first articulated by Rothman [16]. According to this model, a sufficient cause can be made up of multiple components, each of which needs to occur in order for the sufficient condition to occur. For example, in order for a certain bacterium in a certain environment and with a given genetic make-up to acquire antimicrobial resistance both exposure to an antimicrobial drug and external factors such as contact with bacteria carrying a plasmid with resistance against the antimicrobial drug have to occur. In addition, there can be more than one sufficient condition for a cause. For example, another bacterium with a different genetic predisposition or exposed to other environmental conditions may not require exposure to the antimicrobial drug to develop resistance, for instance because of co-selection for heavy-metal resistance. While antimicrobial exposure may be a necessary condition for resistance in one situation, it may not be necessary in all situations, depending on the genetic predisposition and external factors. This model of causation, which is widely accepted by epidemiologists, will be used here. It provides a useful framework for the assessment of causality, including in instances with seemingly contradictory study findings. Moreover, it demonstrates the challenge associated with quantifying the proportion of antimicrobial resistant bacteria associated with different causes: if each cause in fact consists of multiple component causes each occurrence of antimicrobial resistant bacteria could be attributed to each of the component causes.

### Study aims and objectives

The goal of this review is to provide an objective methodical summary of the available scientific evidence for or against a relationship between antimicrobial drug use on farms and antimicrobial resistant human infections. Because this nexus remains contested by some groups, particularly in the U.S., relevant antibiotic use policies and restrictions in the U.S. are highlighted where relevant. To achieve this, we review the scientific evidence supporting or refuting each step in the causal pathways from on-farm antimicrobial use to human public health risk, placing particular emphasis on the strengths and limitations of the available scientific evidence. Breaking down the exceedingly complex pathway from farm to public health risk into discrete intermediary steps considerably reduces complexity and allows for a hypothesis-driven approach. The goal of this study is to characterize the link between antimicrobial use on farms and human infections with resistant bacteria, rather than quantifying the relative importance of this link compared to antimicrobial drug use in other settings even though that quantification will ultimately be important to guide public health policy and data gaps that complicate or prevent quantification are highlighted. Similarly, quantifying the relative importance of different transmission routes from farm to humans is important but beyond the scope of this study.

#### Pathways from antimicrobial drug use on farms to resistant infections in humans

There are different pathways that can lead from antimicrobial use on farms to a public health risk, including foodborne and non-foodborne routes. (Drug residues are a separate public health concern not considered in this study.) Regardless of pathway, four distinct factors have to be understood to characterize the public health risk associated with antimicrobial drug use in animal agriculture.

#### Antimicrobial drug use on farms and feedlots

How antimicrobial drugs are used on farms and feedlots is central to understanding the emergence of antimicrobial resistance. Unfortunately, the amount of information available on actual antimicrobial drug use on farms and feedlots differs considerably across countries and is in many cases insufficient for understanding antimicrobial exposures.

#### Risk of resistance emergence as a result of antimicrobial exposure on farms and feedlots

This question concerns foodborne and zoonotic pathogens as well animal pathogens and commensal bacteria (i.e., the very large number of naturally occurring microorganisms that usually inhabit the body surfaces of humans and animals [17]). Resistance in commensal or animal pathogens poses a human health risk only if the resistance genes can be transferred to human pathogens; this is regardless of whether the transfer occurs in the gut of farm animals, in the environment – be that on farms or off - or within the human gut. Horizontal gene transfer between human commensals and human pathogens has been widely demonstrated [18]. Therefore, evidence of resistance gene transfer from animal-associated bacteria to human commensals can be regarded as indirect evidence for a potential transmission to human pathogens and therefore a public health risk.

#### Risk of infection due to resistant bacteria that emerged on the farm

Bacteria, including foodborne or zoonotic pathogens, can be transferred from food producing animals to humans through direct contact, or indirectly through food or the environment. In addition to contact with the environment in which the animals are raised, indirect transmission may also include exposure to agricultural operations through manure run-offs, airborne particles, or other environmental exposures, even though these transmission routes are considerably less well understood and documented than the other possible transmission routes [19–21]. Some evidence further suggests that humans can transmit bacteria that originated on farms to other humans through direct contact, food contamination during processing, or contamination of shared environments, but, again, the evidence is limited and often circumstantial, the underlying dynamics are not well understood, and at least some animal-associated bacteria may be poorly equipped to be transmitted from human to human [22–24].

In some cases (e.g., foodborne outbreaks) the directionality of infection is quite obvious, but this is not always true. The question of directionality has to be considered in evaluations of the available scientific evidence. However, even if directionality may not always be clear, such studies imply a shared host range, which makes cross-species transmissions likely.

#### Excess morbidity and mortality caused by antimicrobial resistance traits that emerged on farms

Several specific mechanisms by which antimicrobial resistance can have adverse human health impacts have been identified in the literature, of which at least three are directly relevant to resistance that emerged in animal agriculture [25]:

1. Linkage of virulence and resistance traits, leading to drug-resistant strains with increased virulence
2. Treatment delay because initial treatments are ineffective
3. Necessity to choose less desirable treatment options because of resistance to more desirable antimicrobial drugs

Observational studies on the topic can be complicated by the presence of potential confounders such as differences in age or underlying disease among patient groups [26, 27]; the choice of reference group can also have significant impacts on study findings [28]; in addition, bacterial strains can differ in the severity of associated health outcomes independent of antimicrobial resistance [29], thus potentially introducing another source of confounding.

Given the vast amount of literature published on the subject, this review does not strive to be comprehensive in reviewing the available literature associated with the topic. Rather, for each step in the pathway a selected number of studies that exemplify each relevant study type are discussed together with a general discussion of the strength and limitations of the available evidence. Unanswered questions and areas requiring further study are clearly highlighted. Therefore, this study documents the current scientific understanding about the link between antimicrobial drug use on farms and human health risks associated with antimicrobial resistant infections, highlighting what is known and what remains to be determined.

## Main text

### A modified GRADE approach for evaluating the strength of scientific evidence

The approach for evaluating the scientific evidence for a link between antimicrobial use in food producing animals, antimicrobial resistance, and a resulting human health risk is based on the principles guiding the GRADE (Grades of Recommendation, Assessment, Development and Evaluation) approach to systematic reviews. The fundamental notion underpinning this approach is that scientific studies vary in the strength of evidence they provide, or, as stated by the Cochrane Collaboration, the ‘extent to which one can be confident that an estimate of effect or association is close to the quantity of specific interest’. The GRADE approach considers the study type as well as factors relevant to study quality that are specific to an individual study (e.g., width of confidence intervals, inconsistency of results) to arrive at a quality rating for each study. The grade approach considers three basic types of evidence [30]:

1. **Controlled trials** are intervention studies where subjects are allocated by the investigators into treatment and control groups, ideally randomly but occasionally based on another allocation scheme. The GRADE approach classifies randomized controlled trials (RCTs) as ‘high quality of evidence’ because further research is unlikely to change the confidence in the effect estimate. However, as detailed below, the presence of specific study limitations can cause the quality of evidence to be downgraded.
2. **Observational studies** include epidemiological study designs such as cohort, case-control and cross-sectional studies; contrary to RCTs the investigators do not allocate subjects to specific exposures, but compare outcomes among exposed and comparable unexposed population subgroups. The GRADE approach classifies observational studies as ‘low quality of evidence’. Because allocation of individuals to different exposures cannot be controlled, biases are more difficult to control than in RCTs and therefore further research is likely to have an important impact on the confidence in the effect estimate and is likely to change the estimate. The presence of specific study characteristics can cause the quality of evidence to be up- or downgraded.
3. **Any other evidence** is classified in the GRADE approach as ‘very low quality of evidence’ because any effect estimate is uncertain; however, specific study characteristics can cause the quality of evidence to be upgraded, and a variety of study types in this category can provide useful information, including for example molecular subtyping studies, phylogenetic analyses, and case studies or outbreak investigations that lack a proper control group.

In the GRADE approach, the evidence ratings can be up- or downgraded based on a variety of study characteristics that impact study quality, including [31]:

1. **Study limitations** and risk of **bias**
2. **Directness of evidence** (i.e., the extent to which the subjects, interventions and outcome measures are similar to those of interest)
3. **Consistency of results** (i.e., similarity of effect estimates across studies)
4. **Precision of estimates** (e.g., width of confidence intervals around effect estimates)
5. Risk of **residual confounding** and **publication bias**

Finally, the strength of recommendations based on this evidence is affected by [31]:

1. **Quality of evidence**
2. Uncertainty about the **balance between desirable and undesirable effects**
3. Uncertainty or variability in **values and preferences**
4. Uncertainty about whether the intervention represents a **wise use of resources**

Importantly, practical as well as ethical considerations can restrict the type of evidence that can be collected to address a specific research question in a meaningful way [32]. For instance, a randomized controlled trial to evaluate human health outcomes associated with exposure to drug resistant foodborne pathogens would in most cases be unethical.

In the following sections, each step in the causal pathway from antimicrobial use in food producing animals to public health risks caused by infections with antimicrobial drug resistant pathogens is described and the strength of the available evidence is evaluated. A similar approach has been used by the Australian government to review the scientific literature on antimicrobial resistance in animal bacteria and human disease in a study completed in 1999 [33].

Notably, because a number of somewhat different definitions have been used in different scientific disciplines it is important to clearly define the terms ‘antimicrobial drug’ and ‘antibiotic’. For the purpose of his study, and following similar definitions in other authoritative publications [34], we use the term ‘antimicrobial drug’ to refer to ‘antibacterial agents classically used for therapy, prophylaxis, or growth promotion’ as adapted from EFSA [34]. The effects of disinfectants, biocides, antiviral, antifungal or antiparasitic drugs will not be considered while synthetic antibacterial agents will be considered. For the purposes of this study and following the definitions herein, the terms ‘antimicrobial drug’ and ‘antibiotic’ will be used synonymously.

### Evaluating the link between antimicrobial use on farms to the emergence of antimicrobial resistant human infections

#### Antimicrobial drugs use on farms and feedlots

Intervention studies are not applicable for measuring antimicrobial use but a number of observational studies have been conducted. Many follow a cross-sectional design where antibiotic consumption is recorded at one point in time, even though longitudinal designs have also been used and may be preferable to address certain research questions (see for instance [35]). As the examples in Table 1 demonstrate, a number of different data sources are available that contain information regarding on-farm antimicrobial drug use. Data should ideally be based directly on a review of treatment and/or prescription records (e.g., [35–39]). However, this data source may not be readily available or the data collection may be cost-prohibitive. In these cases, more indirect sources may be used, for instance questionnaires administered to veterinarians [40], producers [41–43] or both [44]. In addition, other data sources such as antimicrobial sales data reported by drug manufacturers (e.g., data reported in the U.S. under section 105 of the Animal Drug User Fee Act) may be used albeit the information collected in these cases is less direct and detailed, and consequently less informative. In some cases, it may be possible to collect data from each relevant operation – for instance every producer in the target area (see for instance [36]). Often, however, this is impossible and a subset of operations must be sampled. Ideally, the sampling design should lead to nationally representative estimates (for example [35, 40]). Realistically, it may be possible only to collect data that is representative of a subset of operations, for instance operations representing a segment of the animal agriculture industry such as dairy herds [41] or broiler chicken and turkey flocks [35]. Similarly, studies may focus on operations located in a specific geographic region such as the densest pig production area in Belgium [37], Vietnam’s Tien Giang province [42] or Germany’s state of Northrhine-Westfalia [39]. Regardless of study scope, a robust sampling frame and an appropriate sample size are needed to ensure the study will be representative of the target population, and extrapolations beyond that population may be challenging.

**Table 1:** Examples of select studies available for evaluating antimicrobial consumption in animal agriculture

Reference	Study			Data source	Population; no. of farms or producers or veterinarians	Nationally representative	Directness of evidence	Information regarding			Study timing / period
	Country	Setting	Goals or focus					dosage	duration	reason for use	
GRADE level: observational study											
[35]	France	government	antibiotic consumption metrics	treatment records (reviewed medication purchases, daily records, forms submitted at slaughter; conducted farmer interviews)	Turkey and chicken broiler flocks (*n* = 106; 57 turkey & 49 chicken flocks)	Restricted to western France	actual treatment records	yes	yes	limited	one time
[36]	Denmark	government	antibiotic consumption	treatment records (data collected from pharmacies, veterinarians and feed mills)	all farm animal operations (all prescriptions)	Yes	actual treatment records	yes	yes	yes	annual
[37]	Belgium	academic	antibiotic consumption	treatment records	Pig herds in Belgium’s densest pig production area	Restricted to key pig production area	actual treatment records	yes	yes	limited	one time
[38]	Canada	academic	antibiotic use practices	treatment records & producer questionnaires	Beef herds in western Canada (*n* = 203)	Restricted to western Canada	actual treatment records	yes	yes	yes	one time
[39]	Germany	academic	antibiotic use practices	treatment & prescription records	Cattle farms in Northrhine-Westfalia (*n* = 47)	Restricted to one geographic area	actual treatment records	yes	yes	yes	one time
[40]	Finland	academic	antibiotic use practices	veterinarian questionnaires	Veterinarians in Finland	Yes	response to survey	yes	yes	yes	one time
[41]	U.S.	academic	antibiotic use and farm management practices on conventional & organic operations	producer questionnaire	Dairy farms in 4 U.S. states (*n* = 99 conventional and *n* = 32 organic)	No	response to survey	no	no	yes	one time
[44]	Vietnam	academic	antibiotic use practices and farm management	producer, veterinarian or technical staff questionnaires	Pig and poultry operations in 3 locations in Vietnam (*n* = 270)	Restricted to red river delta	response to survey	no	no	limited	one time
[42]	Vietnam	academic	quantify antibiotic use; identify risk factors for use	producer questionnaire	Chicken farms in Vietnam’s Tien Giang province (*n* = 208)	Restricted to one province	response to survey	yes	yes	limited	one time
[43]	U.S.	government	farm management	producer questionnaire	U.S. operations^a^	Yes	responses to survey; trained interviewer	no	limited	limited	periodic *every 5–7 years*
[45]	U.S.	government	farm financials	producer questionnaire	U.S. operations	Yes	responses to survey; trained interviewer	no	no	no	annual
GRADE level: other											
[129]	U.S.	government	antibiotic sales & distribution	annual reporting of antibiotic sales by drug sponsors	All U.S. drug sponsors	Yes	Sales data	no	no	limited	annual

^a^Survey specific to species and production class (e.g., cow-calf, feedlot); conducted on rotational basis; approximately every 5–7 years

Studies of antimicrobial consumption can be conducted for surveillance or research purposes. Surveillance studies are typically conducted by governmental organizations (e.g., [36, 43]) and research studies more commonly done by academic investigators (e.g., [37–42, 44]). In some cases, a study goal may for instance be to evaluate different antibiotic use metrics (e.g., [35]), but more common research questions involve evaluating antibiotic use practices ([38, 39]) or comparing antibiotic use and farm management practices across different types of operations, such as conventional and organic operations [41]. The study goals have important implications for which study aspects to prioritize if resource limitations force trade-offs among them. For instance, for surveillance purposes it may be more important to collect nationally representative data and to repeat the data collection periodically to evaluate temporal trends ([36, 43, 45]) than to collect highly granular and detailed data on related issues such as correlation of antibiotic use with farm management practices. In contrast, some research questions may require the collection of comprehensive and highly detailed data on study operations but funding may not be available to repeat the study periodically (e.g., [38, 40, 41]). Regardless of purpose, though, information on antimicrobial dosage, duration and reason for use are key determinants of antimicrobial use patterns and necessary to evalaute antibiotic exposures. This information should be collected in surveillance as well as research studies where possible. Unfortunately, this information is not consistently collected in all studies (see Table 1) and for instance in the U.S. this data is currently very scarce.

Antimicrobial sales data are the only nationally representative, annually available data source in the U.S. However, these data do not directly capture antimicrobial use on farms, and are currently not specific to individual species, even though drug companies are required to estimate sales for the major food-producing species (i.e., cattle, swine, chicken and turkey) separately starting with the reporting of the 2016 data. Other nationally representative data sources in the U.S. are surveys conducted as part of the National Animal Health Monitoring System, which are focused on farm management practices, not quantitative with respect to dosage and duration, and currently only conducted every 5 to 7 years; and surveys conducted as part of the Agricultural Resource Management Survey, which are focused on farm finances. Despite these data limitations it is clear that antimicrobial drugs are used on U.S. farms and feedlots. According to the most recent available sales data, almost 10 million kilograms of medically important antibiotics approved for food-producing species were sold in the U.S. in 2015, including 6.9 million kilograms of tetracyclines, 900 thousand kilograms of penicillin and 600 thousand kilograms of macrolides. Also sold were drugs in a number of other classes including aminoglycosides, amphenicoles, cephalosporins, fluoroquinolones, lincosamides, and sulfonamides. While it is thus not currently possible to sufficiently characterize antibiotic exposures on U.S. farms and feedlots they undoubtedly occur.

### Risk of resistance emergence as a result of antimicrobial exposure on farms and feedlots

#### Data based on studies in foodborne and zoonotic pathogens

Data on the emergence of resistance in foodborne or zoonotic pathogens is directly relevant for human health but studies have more commonly focused on commensal bacteria or animal pathogens. Yet, as shown in Table 2, a variety of study types are available that evaluate the link between antimicrobial use on farms and the emergence of resistance in foodborne or zoonotic pathogens. These include controlled trials, various observational study types, and other studies such as microbiological studies comparing resistance levels across production types.

**Table 2:** Examples of studies evaluating the emergence of resistance in foodborne pathogens after food producing animals were exposed to antimicrobial drugs

Reference	Study									Key findings
	Type^a^	species	bacterial target	antimicrobial drug	administration route	study outcomes related to antimicrobial resistance	study design	susceptibility testing	controls	
Grade level: Controlled trial										
[46]	RCT	broiler chicken	*Campylobacter* experimental infection with 5 susceptible strains on day 16 or 24 of life	fluoroquinolones enrofloxacin, sarafloxacin, ciprofloxacin; treated for 5 days, starting on day 30 of life	drinking water	emergence of resistance	2 groups; 25 or 50 chicks per group (half treated)	Phenotypic *agar dilution*	negative controls	resistance emerged rapidly in isolates from treated birds and, for birds treated with enrofloxacin, resistance was retained throughout the sampling period; resistance remained low in untreated controls throughout the study
[47]	RCT	pigs	*Salmonella* Typhimurium DT104; native *E. coli* population monitored; experimental infection with 5 strains isolated from apparently healthy pigs (pentaresistant phenotype)	aureomycin, standard or subtherapeutic dose, treated for 7 days starting 48 h after inoculation	feed	emergence of resistance	3 groups; 6 pigs per group	Phenotypic *agar dilution*	negative controls	resistance emerged in native *E. coli* populations from treated animals and increased resistance persisted for 2 weeks after treatment; treated pigs shed higher numbers of *Salmonella* Typhimurium DT104 than untreated pigs.
[48]	RCT	pigs	*Salmonella* Typhimurium; native *E. coli* population; experimental challenge of piglets 2 days after weaning	aparamycin in different dosing schemes, in some cases followed by sulfamethazine and carbadox or gentamicin and neomycin; treatment initiated 7 days after infection, for 14 days	feed or drinking water	emergence of resistance	2 trials; 6 groups of 8 pigs each	Phenotypic *dilution test*	negative controls	resistance of *Salmonella* isolates was not impacted by treatment regimen; resistance emerged in native *E. coli* populations and was effected by treatment regimen; use of similar antibiotics in rotation resulted in greater resistance than if dissimilar antibiotics were used
[49]	CT	broiler chicken	*Campylobacter;* experimental infection with susceptible strain on day 19 of life	fluoroquinolones: enrofloxacin or flumequine in different concentrations; treated for 4 days, beginning 7 days after infection (or on day 1 of the experiment for one group)	drinking water	emergence of resistance	8 groups; 15 chicks per group	Phenotypic *disc diffusion*	negative controls	resistance emerged in *Campylobacter* isolates from enrofloxacin treated birds (isolates from all other groups remained susceptible)
[50]	CT	beef cattle	*Campylobacter;* naturally occurring bacterial populations	chlortetracycline with or without sulfamethazine; virginiamycin; monensin; or tylosin; treated for 56 days, starting 18 days after arrival at feedlot; antimicrobials removed for 91 days, reintroduced for 42 days	feed	emergence of resistance	30 groups; 10 steers per group	Phenotypic *agar dilution*	negative controls	administration of antimicrobial drugs increased the carriage rate for resistant isolates, but results differed by antimicrobial drug and *Campylobacter* species
[51]	RCT	pigs	*Salmonella* Typhimurium with nalidixic acid resistance; native *E. coli* population; experimental challenge of piglets (from treated or untreated sows)	oxytetracycline, aparamycin; sows treated prior to farrowing	feed	emergence of resistance (in piglets after treatment of sows)	3 groups of pigs	Phenotypic *dilution test* Genotypic *PCR-based*	negative controls	Exposure of sows to oxytetracycline associated with increased resistance in bacterial isolates from pigs
[52]	RCT	Pigs	*Campylobacter;* naturally occurring populations	enrofloxacin; treated with standard therapeutic dose for 5 days	per os	emergence of resistance	2 groups of pigs; 6 pigs per group	Phenotypic *agar dilution*	negative controls	exposure to enrofloxacin resulted in *Campylobacter* isolates resistant to nalidixic acid and ciprofloxacin (no quinolone resistance detected prior to antibiotic exposure); resistant isolates detected for up to 35 days
[53]	RCT	Pigs	*Salmonella* Typhimurium; experimental challenge with marked strain containing nalidixic acid resistance marker	ceftiofur, aparamycin, or carbadox, followed by oxytetracycline; Treated starting 2 days post inoculation	feed (i.m. for ceftiofur)	emergence of resistance	4 groups of pigs; 12 pigs per group	Phenotypic *Disc diffusion*	negative controls	frequency of antimicrobial resistance varied over time – resistance was lowest on day 4 post inoculation but increased steadily afterwards and was highest on the last day of sampling; continued increase of select antimicrobial resistance after exposure was discontinued
Grade level: observational study										
[54]	Cohort period: 2000–2001	dairy cattle	*Salmonella*	various used on conventional farms	n/a	antimicrobial resistance levels	95 farms: 26 organic and 69 conventional	Phenotypic *broth dilution*	organic vs. conventional	results differed by antibiotic; for most antimicrobial drugs, resistance levels were not significantly different; resistance to streptomycin and sulfamethoxazole was greater on conventional farms.
[55]	CS	dairy cattle	*Campylobacter*	various used on conventional farms	n/a	antimicrobial resistance levels	60 farms: 30 organic and 30 neighboring conventional	Phenotypic *disk diffusion*	organic vs. conventional	no statistically significant difference in resistance rates for organic vs. neighboring conventional dairy herds
[56]	CS	broiler chicken and turkey	*Campylobacter*	various used on conventional farms (gentamicin, lincomycin, bacitracin, virginiamycin, etc.)	feed and water	antimicrobial resistance levels	30 farms: 10 organic, 20 conventional	Phenotypic *agar dilution*	organic vs. conventional	antimicrobial resistance rates were significantly higher in isolates from conventional than organic farms, and conventional isolates were significantly more likely to be multi-drug resistant; resistance levels varied across antimicrobial drugs
Grade level: other										
[57]	Longitudinal study	broiler chicken	*Campylobacter*	fluoroquinolones;﻿ difloxacin or enroflaxcin; treatment for 5 days to treat clinically relevant infection; bacterial samples taken before, during and after treatment of flocks	drinking water	prevalence of antimicrobial resistance	5 commercial flocks	Phenotypic *agar dilution* Genotypic *PCR-based*	resistance before, during and after treatment	prevalence of resistant strains increased during treatment and remained elevated after treatment
[58]	bacterial samples at slaughter	broiler chicken	*Campylobacter*	various used on conventional farms	various	prevalence of antimicrobial resistance at slaughter	*n* = 600 ceacal samples from chicken	Phenotypic *dilution method*	resistance levels at slaughter as a function of production type and antimicrobial use history	antimicrobial resistance levels differed significantly by production type and antimicrobial administration history
[59]	bacterial samples of retail meats	Chicken meat	*Campylobacter*	fluoroquinolone	presumably water	Prevalence of resistance	5 producers, meats sampled at retail for 20 consecutive weeks in 2004 and 15 consecutive weeks in 2006	Phenotypic	NAE vs. conventional	No statistically significant difference in fluoroquinolone resistance levels after use was reportedly discontinued (no sample collection or validation at the farm or producer level)
[60]	bacterial samples	chicken and turkey meat; healthy human volunteers	*Enterococci;* VanA-type vancomycin resistant (VRE)	avoparcin use for growth promotion phased out during study period	various	levels of VRE prevalence	convenience samples	N/A	VRE carrier rates before and after the ban	VRE detection in poultry meat and intestinal bacteria of healthy volunteers decreased after the ban
[61]	bacterial samples	humans and poultry products	*Campylobacter*	fluroquinolones	n/a	prevalence of resistance	convenience sample, collected from 1982 to 1989	Phenotypic *disk diffusion*	prevalence of resistance over time	prevalence of quinolone resistance increased from 0 to 14% in poultry products and from 0 to 11% in human samples
[62]	bacterial samples at slaughter	broiler chicken	*Campylobacter*	various used on conventional farms; 1992–1996 time period falls before ban for antibiotic growth promoters, 2001–2002 period does not	various	prevalence of antimicrobial resistance at slaughter	22 flocks; 10 chickens per flock	Phenotypic *agar dilution*	resistance levels at slaughter as a function of production type (free-range or standard) and study period	antimicrobial resistance was more likely in isolates from standard than free-range chicken, differed by *Campylobacter* strain, and for many antimicrobial drugs and strains resistance increased over time
[63]	correlation study	isolates from chicken meat and humans	*Salmonella* Heidelberg, ceftiofur resistant commensal *E. coli*	2003–2008	n/a	prevalence of ceftiofur-resistance in bacterial isolates from chicken meat and human cases	Correlation across provinces and time periods	Phenotypic *broth dilution*	Resistance levels before, during, and after a voluntary discontinuation of ceftiofur use in hatcheries in Quebec	Statistically significant correlation between prevalence of ceftiofur-resistant *Salmonella* Heidelberg on retail chicken and human infections; in Quebec, levels of ceftiofur use in hatcheries during study period seemed correlated with ceftiofur resistance in chicken *Salmonella* and *E. coli* isolates.
[64]	Longitudinal study	pigs	*Enterococcus faecium*	tylosin; resistance to erythromycin, spiramycin, clindamycin, penicillin, tetracycline, chloramphenicol, streptomycin, gentamycin	feed	prevalence of antimicrobial resistance	16 farms; 13 farms were examined again 183 to 287 days later	Phenotypic *disk diffusion*	resistance before and after the ban of tylosin as a growth promoter	Resistance to macrolides, lincosamides and tetracyclines decreased after the use of tylosin for growth promotion was banned.

^a^ *CT* controlled trial (allocation scheme not clearly described), *RCT* randomized controlled trial, *CS* cross-sectional study

Among the controlled trials allocation to treatment or control groups should ideally be random and often is ([46–48]), but this is unfortunately not in all studies sufficiently described [49, 50]. Treatment and control animals are often experimentally challenged with foodborne pathogens such as *Campylobacter* [46, 49] or *Salmonella* [47, 48, 51]. In these cases bacterial strains used for the inoculum are well-characterized and the emergence of resistance can easily be detected. However experimental challenges may differ from naturally occuring infections in important ways including for instance the types and diversity of bacterial strains used for infection, the metabolic profiles of the bacteria at the time of infection, and the bacterial quantities used for challenge. Naturally occuring bacterial populations of *Campylobacter* have also been investigated [50], and in some cases naturally occuring populations of *E. coli* have been analyzed together with experimentally inoculated human pathogens [48, 51]. In most cases, the outcome of interest is phenotypic resistance measured by dilution or, more rarely, disc diffusion test but phenotypic methods have also been combined with genotyping, which provides more detailed information about the emerging resistance traits [51].

A number of controlled studies have demonstrated the emergence of resistance in *Campylobacter* isolates after exposure to antimicrobial drugs . For instance, one study experimentally infected groups of 15 broiler chicken each on day 19 of life with a quinolone-sensitive *Campylobacter* strain, and exposed the birds to enrofloxacin flumequine at low (15 ppm) or high (50 ppm) dosages in drinking water for 4 days, beginning on day 26 of life, 7 days after infection [49]. On days 29, 33 and at slaughter on day 43, *Campylobacter* isolates from both exposed groups harbored resistance to enrofloxacin as well as nalidixic acid and flumequine, while those from unexposed control groups remained sensitive to these drugs and non-infected control groups did not yield *Campylobacter* isolates, making introduction of other *Campylobacter* strains from outside sources unlikely. In another trial, broiler chicken were experimentally infected with susceptible *Campylobacter* strains on days 16 or 24 of life and exposed to enrofloxacin or sarafloxacin in drinking water for 5 days, starting on day 30 of life [46]. Fluroquinolone resistance emerged rapidly in *Campylobacter* isolates from exposed birds, and, for birds treated with enrofloxacin, resistance was retained throughout the sampling period. Another controlled trial evaluated the impact of a single enrofloxacin exposure on naturally occuring *Campylobacter* populations isolated from pigs. Before exposure, all 867 collected isolates were susceptible to nalidixic acid and ciprofloxacin. After treatment, isolates with high resistance to nalidixic acid and ciprofloxacin were detected in the exposed groups, up to 35 days after treatment [52]. Similar observations have also been made for naturally occuring *Campylobacter* populations in beef cattle. In this study, steers were exposed to a variety of antimicrobial treatments (i.e., chlortetracycline with or without sulfamethazione, virginiamycin, monensin and tylosin) in feed for 56 days, and then again for 42 days after 91 days without any antimicrobial exposures [50]. Exposure to antimicrobials resulted in an increased carriage rate for resistant *Campylobacter* isolates but the authors noted marked differences among antimicrobial drugs and *Campylobacter species*. For *Campylobacter jejuni*, administration of antimicrobials significiantly increased resistance rates to ampicillin and tetracycline but not erythromycin. In contrast, for *Campylobacter hyointestinalis*, results were not statistically signficiant for ampicillin and erythromycin resistance was statistically signficiant only in cattle fed tetracycline, highlighting the potentially important differences among antimicrobial drugs and bacterial species.

In fact, studies for *Salmonella* have yielded somewhat different results. A randomized controlled trial investigating the impact of different treatment regimen administered to pigs through feed or drinking water failed to find a statistically significant impact of antimicrobial exposure on resistance in the *Salmonella* Typhimurium isolates used in the experimental challenge, but did find an impact on resistance in the native *E. coli* population which was effected by treatment regimen [48]. In that study, piglets were experimentally challenged with *Salmonella* two days after weaning and treatement through feed or drinking water was initiated 7 days after infection, for 14 days. Notably, data for *Salmonella* Typhimurium were not actually presented in the study and isolation rates were not reported. Still, the reasons for this apparent difference in results for *Salmonella* compared to native *E. coli* are not clear. In another controlled trial, sows were treated with oxytetracycline prior to farrowing (or not), their piglets challenged with *Salmonella* Typhimurium, and then piglets were treated with oxytetracycline or apramycin (or not at all) [51]. Resistance levels in the *Salmonella* isolates were not significantly effected by the treatment regimen, but resistance to both aparamycin and oxytetracycline was reportedly highest in *E. coli* isolates from pigs that nursed sows treated with oxytetracycline prior to farrowing. In yet another randomized controlled trial, piglets were experimentally infected with *Salmonella* Typhimurium and exposed to aureomycin fourty-eight hours after challenge. Exposed pigs consistently shed statistically significantly higher numbers of *Salmonella* than unexposed controls but resistance was not reported for *Salmonella* isolates. On the contrary, in another randomized controlled trial pigs were experimentally challenged with a *Salmonella* Typhimurium strain carrying a nalidixic acid resistance marker and then exposed to ceftiofur and oxytetracycline, aparmycin and oxytetracycline, or carbadox and oxytetracycline [53]. Resistance levels varied over the study period and differed by antibiotic treatment but for all treatments were highest on the last day of the study. Notably, for aparamycin, carbadox and ceftiofur, resistance continued to increase after the antimicrobial exposure was discontinued.

Evidence from observational studies supports a risk of resistance emergence in foodborne pathogens exposed to antimicrobial drugs on farms but also reinforces the complexity of the issue. For instance, one cohort study during the years 2000 and 2001 compared phenotypic resistance to various antimicrobial drugs in *Salmonella* isolates from 69 conventional and 26 organic daries. Results differed by antibiotic. Resistance to streptomycin and sulfamethoxazole was greater on conventional farms while for most antimicrobial drugs, resistance levels were not significantly different [54]. In contrast, a cross-sectional study of *Campylobacter* resistance in insolates from 30 organic dairies and 30 neighboring conventional operations failed to find a statistically significant difference in resistance rates [55]. Yet another cross-sectional study of *Campylobacter* resistance in conventional and organic broiler chicken and turkey flocks found signficiantly higher antimicrobial resistance rates in isolates from conventional compared to organic farms [56]. In addition, isolates from conventional farms were significantly more likely to be multi-drug resistance, and resistance levels varied across drugs.

A variety of other study designs have also been used to investigate the issue and generally support a risk of resistance emergence after exposure, even though the strength of evidene is significantly lower compared to that from controlled trials or observational studies. For instance, one study investigated the prevalence of antimicrobial resistance in *Campylobacter* isolates from 5 commercial broiler flocks before, during and after they were exposed to difloxacin or enrofloxacin in the drinking water [57]. Even though this study lacks proper control groups it was conducted in commercial flocks and therefore provides a valuable complement to the controlled trials discussed above. The prevalence of resistant strains increased significantly during treatment and remained elevated after treatment. Other studies have compared rates of resistance across production systems [58, 59], over time periods that coincided with the introduction or ban of certain antimicrobials [60, 61], or both [62]. These studies generally support a risk of antimicrobial resistance emergence after on-farm exposure. A drop in *vanA* –type vancomycin resistance in *Enterococci* from poultry meat and health volunteers, for instance, coincided with a ban of avoparcin as growth promoter [62], ceftiofur resistance levels in *Salmonella* Heidelberg from retail chicken and human cases decreased while ceftiofur use in hatcheries was temporarily discontinued and increased when it was reinstated [63], and a rise in fluoroquinolone resistant *Campylobacter* coincided with the introductin of enrofloxacin [61]. Notably, another study failed to find a statistically significant drop in fluoroquinolone resistance among *Campylobacter* isolates after their use was supposedly discontinued [59].

Taken together, these studies provide evidence that antimicrobial use on farms can and does lead to an increase in resistance among foodborne pathogens isolated from the exposed animals. How quickly resistance emerges seems to depend on many factors, including the pathogen species, strain and drug treatment of interest. In some instances, discontinuation of use may lead to a rapid decrease in resistance; for example, a decrease in vancomycin-resistant *Enterococci* (VRE) coincided with a ban of avoparcin as growth promoter [60], and a decrease in vancomycin resistance was demonstrated on individual farms after the ban of antimicrobial growth promoters [64]. In other cases, however, resistance levels have remained increased for extended periods of time after antibiotic uses were discontinued. For example, in a longitudinal study of farms that discontinued fluoroquinolone use as well as control farms, resistance rates in *Campylobacter* isolates remained high for years after use was discontinued [59]. Similarly, vancomycin resistance in *Enterococci* isolates from pigs did not decrease in response to a discontinuation of use until the use of tylosin, a macrolide, was also discontinued, because both resistance traits were genetically linked [2]. Also notably, antimicrobial exposure does not always lead to a statistically significant increase in resistance rates during the studied time periods [54]. Similarly, resistance levels in bacteria associated with operations that do not use antimicrobial drugs are not in all cases significantly lower than those associated with operations that do [55]. Therefore, while exposure of foodborne pathogens to antimicrobial drugs on farms or feedlots clearly poses a risk of resistance emergence that risk is currently difficult to quantify.

#### Data based on studies in animal pathogens or commensal bacteria

Bacteria can readily share their genetic material. The emergence of antimicrobial resistance in commensal bacteria or animal pathogens contributes to the abundance of resistance genes that may subsequently be transferred to human pathogens. Because commensal bacteria are ubiquitous in the environment and their study tends to be easier than that of foodborne or zoonotic pathogens, considerably more studies have focused on the impact of antimicrobial exposure on commensal bacteria than foodborne or zoonotic pathogens. Only a few representative studies are highlighted in Table 3, as illustrative examples. Additional examples are provided in other reviews, including that performed by the Joint Expert Technical Advisory Committee on Antibiotic Resistance (JETACAR) [32].

**Table 3:** Examples of study types evaluating the emergence of antimicrobial resistance in natural bacterial background populations after food producing animals were exposed to antimicrobial drugs

Reference	Study									Key findings
	Type^a^	species	bacterial target	antimicrobial drug	administration route	study outcomes related to antimicrobial resistance	study design	susceptibility testing	controls	
Grade level: Controlled trial										
[65]	RCT	cattle	*E. coli*	chlortetracycline (with or without sulfamethazine), monensin, tylosin, virginiamycin; all as growth promoters	feed	emergence of antimicrobial resistance	6 treatments, 5 pens per treatment, 10 steer per pen	Phenotypic agar dilution	negative control	exposure to chlortetracycline and sulfamethazine increased the prevalence of resistance; diet had an important impact on shedding of resistant strains
[66]	RCT	cattle	*E. coli*	florfenicol	s.c. injection	emergence of antimicrobial resistance	42 pens (8–10 cows per pen), 2 cows per pen enrolled in study	Phenotypic *disk diffusion*	negative control	after antimicrobial treatment, increased antimicrobial resistance; prior treatment predictive of resistance; animal source and previous management impact results
[67]	RCT	Cattle	fecal bacteria	ceftiofur, tetracycline	s.c. injection	antimicrobial resistance	176 steers; 2 replicates of 88 steers each, 8 pens of 11 steers per replicate; total of 4 treatment groups	Genotypic *PCR based*	positive and negative controls	Initial and subsequent antimicrobial treatments led to increased frequency of resistance genes
[68]	RCT	pigs	*Enterococci Staphylococcus hyicus*	tylosin (as growth promoter)	feed	erythromycin resistance levels	2 trials a 10 pigs, *n* = 5 per group	Phenotypic	negative control	Prevalence of resistance in enterococci immediately increased 2.4× in response to tylosin exposure; resistance in *Staphylococcus hyicus* occurred more gradually, at a rate of 8% per day and 5× over 20 days
[130]	RCT	pigs	*aerobic gram negative bacteria; Salmonella*	chlortetracycline	feed	antimicrobial resistance	3 farms (convenience sample); 12, 2 and 8 barns per farm	Phenotypic *broth dilution*	negative control	antimicrobial exposure was associated with increased prevalence of resistance in aerobic gram-negative bacteria
GRADE level: observational study										
[69]	cohort	pigs	coliform bacteria	olaquindox	feed	emergence of resistance	12 farms	Phenotypic	neighboring farms that do not use olaquindox	prevalence and level of resistance increased on farms that used it, and to lesser extent on adjacent farms
[70]	CS	pigs	*E. coli*	various	feed	emergence of antimicrobial resistance	34 farms	Phenotypic	negative control	antimicrobial use increases the emergence of resistance
[71]	CS	pigs	*E. coli*	various	various	antimicrobial resistance	47 farms	Phenotypic	negative control	antimicrobial use (in weaner rather than finisher pigs) was associated with resistance; some evidence for cross- resistance and co-selection
GRADE level: other										
[72]	correlation study	pigs, cattle, poultry	*E. coli*	various	various	correlation between antimicrobial use and resistance	7 countries	phenotypic	comparison across countries	use of antimicrobial drugs is strongly correlated with resistance in *E. coli*
[73]	correlation study	Poultry	*Enterococci, Vancomycin-resistant*	avoparcin	various	Comparison of resistance prevalence before and after avoparcin ban	Italy	phenotypic	comparison over time	prevalence of vancomycin-resistant *Enterococci* decreased in poultry meat samples after the avoparcin ban

^a^ *CT* controlled trial (allocation scheme not clearly described), *RCT* randomized controlled trial, *CS* cross-sectional study

As shown in Table 3, a variety of study types are available that investigate the emergence of resistance in commensal bacteria in response to antimicrobial exposure. These include controlled trials, observational studies, and correlation studies that compare bacterial populations across settings. Similar to the situation described in the preceding section, most studies rely on phenotypic methods to evaluate resistance levels, and studies vary vastly in the amount of control they exert on treatment allocation and antimicrobial exposures. Because the primary focus is on commensal bacteria, studies typically follow naturally-occurring bacterial populations, even though, as described in the preceding section, in some cases animals are experimentally challenged with pathogens and both pathogens and commensals are followed over time.

A number of randomized controlled trials have demonstrated an increase in the prevalence of antimicrobial resistant bacteria following antimicrobial exposure. For instance, in one study feedlot steers were fed chlortetracycline with or without sulfamethazine, monensin, tylosin or virginiamycin for 61 days in a silage-based diet, followed by 86 days without antimicrobials, and then another 42 days of exposure while being on a grain-based diet [65]. Expsoure to antimicrobial drugs significantly incrased the prevalence of steers shedding resistant baceria and the data suggested a potential genetic link between tetracycline and ampicillin resistance. Notably, the type of feed significantly impacted shedding rates with higher rates associated with the grain-based diet, emphasizing the importance of external factors. In another randomized controlled trial, feedlot steers were exposed to a single dose of florfenicol by subcutaneous injection and resistance in fecal *E. coli* populations were measured before and immediately after exposure, and up to a month later [66]. Notably, treated and control animals were housed in the same pen together with other steers. Immediately after treatment, all isolates from treated animals were resistant to multiple antimicrobial drugs including chloramphenicol, and resistance persisted in later samplings. The source of the animals and their previous management were found to have a statistically significant impact on the resistance dynamics. Yet another study used a metagenomic approach [67]. Feedlot steers were exposed to chlortetracycline – administering the drug either to one or all steers in the pen – followed by administration of chlortetracycline (or no antimicrobial drugs). Fecal bacterial communities were analyzed for common resistance genes (*i.e.*, bla_CMY-2_, bla_CTX-M_, tet(A) and tet(B)) by real-time PCR. A determination of the bacterial species carrying the resistance genes was not performed but 16S rRNA gene quantity was determined to standardize the number of resistance gene copies. The frequency of ceftiofur resistance genes increased significantly when all steers received ceftiofur while tetracycline resistance decreased at the same time, likely indicating a shift in the composition of the microbial community. In response to subsequent chlortetracycline exposure both ceftiofur and tetracycline resistance genes increased.

Similar results have also been observed in pigs. In one trial, for instance, pigs on 3 farms were exposed to chlortetracycline in the feed and resistance was evaluated in their aerobic gram negative bacteria as well as *Salmonella*. Antimicrobial exposure was associated with increased prevalence of resistance in aerobic gram-negative bacteria. In another study, pigs were fed tylosin and weekly samples were taken to evaluate macrolide resistance in fecal enterococci and skin staphylococci [68]. The prevalence of resistance in enterococci immediately increased 2.4 times in response to tylosin exposure. By comparison, resistance in *Staphylococcus hyicus* occurred more gradually, at a rate of 8% per day and 5 times over 20 days.

As shown in Table 3, there is also strong evidence from observational studies as well as the analysis of convenience samples that antimicrobial use on farms leads to an increase in resistance among commensals and animal pathogens isolated from the exposed animals. In one cohort study, for instance, the impact of olaquindox exposure on coliform bacteria from pigs was analyzed and compared to resistance in coliform bacteria from pigs on neighboring farms not exposed to olaquindox [69]. Both the prevalence and level of resistance increased on farms that used olaquindox, and to lesser extent on adjacent farms. Two cross-sectional studies of 34 and 47 pig farms provide further evidence for a risk of resistance in response to antimicrobial exposure [70, 71]. Notably, one of the studies provided some evidence for co-resistance and cross-selection, and noted that antimicrobial use in weaners was associated with incrased resistance [71]. In addition, a study of *E. coli* convenience samples across 7 countries revealed a correlation between antimicrobial use and resistance [72] and an analysis of *Enterococci* from poultry samples collected before and after the avoparcin ban found the prevalence of vancomycin-resistant *Enterococci* decreased in poultry meat samples after the avoparcin ban [73].

Taken together there is ample evidence supporting a risk of resistance emergence in commensal bacteria after exposure to antimicrobial drugs. How quickly resistance emerges is affected by a variety of factors, including the bacterial species and the antimicrobial treatment choice as well as potential preceding antimicrobial exposures [66, 68]. A variety of external factors, such as the type of feed chosen, can also influence the emergence of resistance [65]. Resistance may be reversible [73], but can remain at elevated levels after use is discontinued [47], and a single exposure can lead to increased resistance in the commensal bacterial population [66]. Therefore, while exposure to antimicrobial drugs on farms or feedlots clearly poses a risk of resistance emergence in commensal bacteria that risk is currently difficult to quantify.

#### Data based on studies in humans, laboratory animal models or in vitro systems

Data on the correlation between antimicrobial use and the emergence of antimicrobial resistance have also been collected in human healthcare, laboratory animal models and in vitro systems. Results collected in these settings are generally consistent with the observations made in agricultural settings. As shown in Table 4, a variety of study types are available, including RCTs, observational studies, and other types of evidence. Many of the scientific studies have analyzed the emergence of resistance in humans during treatment, monitoring the resistance of bacteria isolated from the patients over time. In some cases, patients were randomly allocated to treatment arms, whereas other studies compared isolates from patients that received treatments with different antimicrobial drugs or treatment regimen. Yet other studies followed bacterial populations in laboratory animals or in vitro systems, or evaluated correlations between antibiotic use practices and the prevalence of resistance.

**Table 4:** Examples of studies evaluating the emergence of antimicrobial resistance in response to antimicrobial exposure

Reference	Study							Key findings
	Type^a^	study population	drug of concern	bacterial target	endpoint or outcome relevant to antimicrobial resistance	controls	period of concern^b^	
Grade level: Controlled trial								
[74]	SR of RCTs; *n* = 46 studies	pneumonia patients	imipenem	*Pseudomonas aeruginosa*	initial resistance; resistance emergence on treatment	other antimicrobials: beta-lactams, aminoglycosides, vancomycin, fluoroquinolone	1993–2008	resistance emerged: imipenem: 38.7% (range: 5.6–77.8%) of isolates comparator groups: 21.9% (range: 4.8–56.5%)
[75]	MA of RCTs; *n* = 8 studies	patients with various infections	beta-lactam	various	emergence of antimicrobial resistance	other antimicrobials: beta-lactam and aminoglycoside combination	1980–2004	resistance emerged in various bacterial species (up to 20% of isolates); no significant difference between the two treatment arms in resistance emergence
[85]	RCT; *n* = 313 patients	patients with nosocomial pneumonia, sepsis, or severe diffuse peritonitis	imipenem	*Pseudomonas aeruginosa*	emergence of antimicrobial resistance	imipenem plus netilmicin	1988–1992	resistance to imipenem occurred in monotherapy (*n* = 8) and combination therapy (*n* = 13) patients
Grade level: Observational study								
[76]	SR & MA of RCTs and OS; *n* = 24 studies	patients with various infections and healthy volunteers	various	various	resistance emergence under treatment	other antimicrobials	1955–2009	prescription of antibiotics in primary care is associated with resistance in bacteria
[77]	cohort study; *n* = 271 patients	hospitalized patients with lab-confirmed *Pseudomonas* infection where initial isolate was susceptible	ceftazidime ciprofloxacin imipenem piperacillin	*Pseudomonas aeruginosa*	emergence of resistance during treatment	compare patients with resistance emergence to those without	1994–1996	resistance emerged in 10% of patients (*n* = 28); risk differed by antimicrobial drug
[78]	cohort study; *n* = 2641 patients	coronary artery bypass graft surgery patients	Various; short vs. prolonged antibiotic prophylaxis	*Enterobacteriaceae* (cephalosporin resistant); *Enterococci* (vancomycin resistant)	emergence of resistance during prophylaxis	compare patients with acquired antibiotic resistance to those without	1993–1997	Prolonged antibiotic prophylaxis was associated with an increased risk of acquired antibiotic resistance
[79]	CC study	patients with lab-confirmed bacteremia, resistant *Pseudomonas*	Piperacillin, ceftazidime, imipenem, ciprofloxacin, gentamicin, amikacin	*Pseudomonas aeruginosa*	odds of previous antibiotic treatment	patients with lab-confirmed bacteremia caused by susceptible *Pseudomonas* strains	1989–1998	previous exposure to antimicrobial monotherapy is associated with increased risk of subsequent resistance to that antimicrobial
Grade level: Other								
[80]	in vivo trial	mice experimentally challenged with bacteria	amikacin, ceftriaxone, perfloxacin or their combination	*Pseudomonas aeruginosa*, *Klebsiella pneumoniae*, *Enterobacter cloacae*, *Serratia marcescens*	resistance emergence after treatment	other antimicrobials (single vs. combination therapy)	1986	resistance emerged in response to treatment; the frequency of resistance emergence varied by pathogen and antibiotic / combination
[81]	in vitro trial (lab evolution system)	bacteria experimentally exposed to antimicrobial	colistin (different treatment regimen)	*Acinetobacter baumannii*	resistance emergence after exposure	negative control	2007	extensive resistance emerged during re-growth (as early as 6 h after treatment)
[82]	Cross-national database study (26 countries in Europe)	antibiotic use in outpatient settings (calculated as defined daily dose per 1000 inhabitants) and antibiotic resistance rates	various	various	correlation between antibiotic use and resistance across countries	n/a	1997–2002	rates of antibiotic resistance are higher in high consuming countries

^a^ *SR* systematic review, *MA* meta-analysis, *RCT* randomized controlled trial, *OS* observational study, *CC* case-control study

^b^For experimental laboratory studies that do not specify the date of the experiments the publication date is substituted

A systematic review of randomized controlled trials in pneumonia patients with initially sensitive *Pseudomonas aeruginosa* infection, for instance, found that resistance emerged readily *de novo* in response to imipenem exposure, with an average of 38.7% of isolates (range: 5.6–77.8%) acquiring resistance [74]. Notably, *de novo* resistance emergence was less likely in the comparator group treated with other antimicrobials (i.e., beta-lactams, aminoglycosides, vancomycin, or fluoroquinolone), where resistance emerged in an average of 21.9% of isolates (range 4.8–56.5%). Another meta-analysis of human patients with various types of infections treated with beta-lactam antibiotics or a combination of beta-lactams and aminoglycosides found that resistance rapidly emerged *de novo*, with up to 20% of isolates becoming resistant, and there was no statistically significant difference between the two treatments [75]. Results from a variety of observational studies as well as meta-analyses of individual observational studies also support a risk of resistance emergence in response to antimicrobial exposure [76–79]. Studies in laboratory animals and in vitro models have also demonstrated for a number of different bacteria and drugs that antimicrobial resistance readily emerges in response to exposure, sometimes very quickly, even though the dynamics of that resistance depend on the specific bacterium and drug [80, 81]. Similar to the situation in animal agriculture [72], a cross-national study has demonstrated a correlation between antibiotic use patterns and resistance levels, showing that rates of antimicrobial resistance are higher in high-consuming countries [82].

Together, these data further support that antimicrobial drug use can and does lead to the emergence of resistance in exposed bacteria. The data also suggest that in general, longer exposure periods and preceding antimicrobial exposure tend to be associated with an increased risk of resistance compared to short exposure times [78, 79, 83]. However, how quickly resistance emerges depends on the pathogen, drug, and resistance mechanism, among other factors [80, 83]; exposure to a drug can also select for resistance to a related drug due to cross-selection, and some resistance may be maintained in the absence of antimicrobial selection, for instance due to co-selection [84]. The data further suggest that certain treatment regimens may be associated with an increased risk of resistance compared to others (see for instance [77, 78, 85]), but more data are needed to allow the optimization of treatment regimen to minimize the risk [83, 86] and data specific to animal agriculture are needed to determine how to translate the information to optimize treatments in this regard in veterinary settings.

### Risk of infection due to resistant bacteria that emerged on the farm

#### Transmission of foodborne or zoonotic pathogens to humans

Because of ethical considerations, RCTs that evaluate the risk of pathogen transmission from animals to humans are generally not feasible. However, there is considerable evidence from observational studies, as well as other study types, such as outbreak investigations, case reports, and bacterial studies of correlation among bacteria from different sources that foodborne or zoonotic pathogens carrying antimicrobial resistance genes are shared between animals and humans (Table 5). Many of the studies have focused on *Salmonella* or methicillin-resistant *Staphylococcus aureus* (MRSA), and many are based on case-control studies that compare risk factors for infections with resistant or susceptible isolates retrospectively. In at least some studies, the directionality of transfer remains unknown; and for at least some pathogens and settings, cross-species transfers may be relatively infrequent. Moreover, in some instance the study analyzed colonization with a potential human pathogen such as MRSA, rather than actual infection that led to clinical disease. While colonization with these potential pathogens clearly demonstrates transmission, the associated public health risk is less clear.

**Table 5:** Examples of study types evaluating the human health risk posed by infections with antimicrobial resistant foodborne or zoonotic pathogens

Reference	Study									Key findings relevant to antimicrobial resistance
	Type^a^	subjects	country	time period	bacterial target	relevant study outcomes	sample size	controls	comments	
GRADE level: Controlled trial										
Not applicable because of ethical concerns										
GRADE level: observational study										
[87]	CC	human patients	U.S.	1999–2001	*Salmonella* Newport; MDRAmpC resistant	risk factors for infection	34 cases; 37 infected controls and 94 community controls	*Salmonella* patients with susceptible Newport infection; healthy community controls	followed up by investigation of cattle isolates and risk factors for shedding on dairy farms; regional in scope (New England)	Association of infection with *Salmonella* Newport MDRAmpC with dairy farm exposure; bacterial isolates from humans and cattle were indistinguishable or closely related (based on antibiotic resistance profile and PFGE pattern)
[88]	CC	human patients	U.S.	2002–2003	*Salmonella* Newport; MDRAmpC resistant	risk factors for infection	215 case patients; 54 with MDRAmpC resistant strain and 146 pansusceptible; 1154 community controls	healthy community controls; volunteers without diarrhea in the previous 28 days	FoodNet study; national in scope (8 of 9 FoodNet sites)	Association of infection with *Salmonella* Newport MDRAmpC with consumption of uncooked ground beef or home prepared runny scrambled eggs or omelets during the 5 days before onset of illness
[89]	CC	human patients in Wisconsin	U.S.	2003–2005	*Salmonella* Newport MDR AmpC	associations between antimicrobial resistance and reported exposures	268 isolates from Wisconsin and 402 from the remaining U.S.	Cases in Wisconsin vs. rest of the U.S.	Controls were collected 2003–2004	Infections with multidrug-resistant *Salmonella* Newport strains were associated with exposures to cattle, farms and unpasteurized milk
[90]	CC	human patients in California	U.S.	1985	*Salmonella* Newport (chloramphenicol resistant)	risk factors for infection	45 patients and 89 matched controls	healthy volunteers	Epidemic *Salmonella* strain was isolated from hamburger products eaten by cases, as well as the abattoirs where the animals were slaughtered, dairies that sent the animals to slaughter, and ill dairy cows.	Association of infection with resistant *Salmonella* Newport isolates with eating ground beef during the week before onset and penicillin and tetracycline use during the month before onset of symptoms; chloramphenicol resistant *Salmonella* from dairy farms were associated with chloramphenicol use on those dairies
[91]	CC	human patients from national database	Netherlands	2003–2005	MRSA (non-typable)	risk factors for infection	35 cases, 76 controls	MRSA infections with other strains	Analysis aimed to exclude secondary cases	Living in rural areas and contact with pigs and cattle were associated with case patients
[92]	CS	veal calf farmers	Netherlands	2007–2008	MRSA ST 398	risk factors for carriage	102 farms, 390 questionnaires, 2151 nasal swabs from calves	farmers negative for MRSA ST 398	nasal swabs taken from farmers and farm calves; farms randomly selected	Human MRSA ST 398 carriage was associated with intensity of animal contact and number of positive animals on farm; calves were more likely to be carriers when treated with antibiotics, and good farm hygiene had a protective effect.
[93]	CS	swine farmers	Belgium	2007	MRSA ST 398	risk factors for carriage	49 swine farms, 127 persons on the farms participated and 1500 pigs were sampled	farmers negative for MRSA ST 398	nasal swabs and wound samples; farms randomly collected	MRSA ST 398 carriage by farmers was associated with prevalence among pigs on the farm, having regular contact with pigs, dogs, and horses, and use of protective equipment.
GRADE level: other										
[22]	phylogenetic study	bacterial isolates	various	1997–2011	MRSA CC398	evolution and transmission dynamics including transmission between species	n/a	none	Phylogenetic and population genetic analysis	human and animal populations of CC398 are separate, but with multiple transfers back and forth; same data suggesting onward transmission of animal-associated strains in community settings (e.g., hospitals); different tetracycline resistance genes showed different evolutionary patterns
[94]	outbreak	human patients with rare strain	Denmark	1998	*Salmonella* Typhimurium DT104	source of multidrug resistant infection outbreak	27 cases	none	rare strain isolated from patients and pork samples; pork samples were traced back to herd; resistant infections in humans were more difficult to treat	A swine herd was the likely source of a human outbreak with MDR *Salmonella* Typhimurium DT104
[95]	outbreak	human patients infected with rare strains	U.S.	1983	*Salmonella* Newport	source of multidrug resistant infection outbreak	18 cases	none	rare isolate strain found in human patients and cattle	hamburger from beef cattle fed sub therapeutic chlortetracycline for growth promotion likely outbreak source
[96]	human case report	human patient infected with rare strain	U.S.	1998	*Salmonella* Typhimurium	source of multidrug resistant infection	1 case	none	Infection traced back to farm;	The farm was identified as the source of a multidrug resistant *Salmonella* isolate from a human case patient
[97]	human case report	MRSA cluster around pig farmer	Netherlands	2004	MRSA	source of multidrug resistant infection	pig farmer, family and co-workers, pigs	none	animals on farm sampled	Infection with MRSA traced back to farm, onward transmission in farm family
[98]	correlation study	Isolates from swine and swine workers	U.S.	Unspecified	MRSA	risk factors for MRSA colonization	20 workers and 299 swine	none	animals and humans sampled	Colonization with MRSA ST 398 was very common in swine herds, similar isolates in humans and animals
[99]	correlation study	isolates from farm personnel and milk samples	Hungary	2002–2004	MRSA	risk factors for MRSA colonization	1 farm, 12 workers sampled, 595 milk samples	none	MRSA strains were subtyped	MRSA isolates from workers in close contact with cows were indistinguishable from cow isolates

^a^ *CC* case-control study (or case-case study where controls are cases of infection with other strains), *CS* cross-sectional study

A number of observational studies have evaluated the human health risk posed by infections with antimicrobial resistant foodborne or zoonotic pathogens. A retrospective case-control study in New England (U.S.), for instance, evaluated risk factors for infections with a multidrug-resistant *Salmonella* Newport strain (MDR AmpC), comparing 34 case patients to 37 controls infected with susceptible *Salmonella* Newport strains and 94 healthy community controls [87]. Infections with the multidrug-resistant *Salmonella* Newport strain were significantly associated with dairy farm exposure.

(Adjusted odd ratio 12.2; 95% Confidence Interval: 1.2–640). Investigation of two regional dairy farms where the *Salmonella* Newport strain had recently been identified detected the strain in animals and ill farm workers. In fact, bacterial isolates from humans and cattle were indistinguishable or closely related based on antibiotic resistance profiles and PFGE patterns. Another case-control study, also focusing on *Salmonella* Newport MDR AmpC in the U.S. but national in scope and comparing 54 case patients to 146 controls infected with pan-susceptible *Salmonella* Newport strains and 1154 healthy community controls, identified consumption of uncooked ground beef or home prepared runny scrambled eggs or omelets during the 5 days prior to illness onset as a significant risk factor [88]. A third case-control study of *Salmonella* Newport MDR AmpC, comparing 268 cases from Wisconsin to 402 controls from the remaining U.S. found infections with *Salmonella* Newport MDR AmpC significantly associated with exposure to cattle, farms and unpasteurized milk [89]. Yet another case-control study of 45 case patients infected with *Salmonella* Newport strains carrying chloramphenicol resistance and 89 matched healthy controls identified eating ground beef during the week before illness onset and penicillin and tetracycline use during the month before onset of symptoms as significant risk factors [90]. Notably, the same *Salmonella* strain was isolated from hamburger products eaten by case patients, as well as the abattoirs where the animals were slaughtered, dairies that sent the animals to slaughter, and ill dairy cows. Chloramphenicol resistant *Salmonella* from dairy farms were associated with chloramphenicol use on those dairies. In addition, case-control [91] and cross-sectional [92, 93] studies in the Netherlands and Belgium have identified animal contacts as risk factors for carriage or infections with MRSA. In one study of veal calf farmers in the Netherlands, human MRSA ST 398 carriage was associated with intensity of animal contact and number of positive animals on farm; calves were more likely to be carriers when treated with antibiotics, and good farm hygiene had a protective effect [92]. In another study of swine farmers in Belgium, MRSA ST 398 carriage by farmers was associated with prevalence among pigs on the farm, having regular contact with pigs, dogs, and horses, and use of protective equipment [93]. A case-control study of human infections with non-typable MRSA in the Netherlands, comparing 35 cases to 76 controls based on a national database of human patients, identified living in rural areas and contact with pigs and cattle as significantly associated with case patients [91].

In addition, *Salmonella* outbreaks have been traced back to farm animals. For instance, an outbreak of multidrug-resistant *Salmonella* Typhimurium DT104 that occurred in Denmark in 1998 was traced back to a swine herd [94]. The outbreak strain was a rare strain, simplifying the trace-back. In fact, the same rare strain was isolated from patients and pork samples and pork samples were traced back to the swine herd of origin. Notable, the resistant infections in humans were more difficult to treat due to the resistance profile. In another outbreak involving a rare strain of *Salmonella* Newport that occurred in the U.S., hamburgers from beef cattle fed chlortetracycline were identified as the likely outbreak source [95]. Trace-back investigations revealed the same rare *Salmonella* strain found in human patients was shed by cattle. Human case reports have identified farm exposures as the likely source of infections with multidrug resistant *Salmonella* Typhimurium in the U.S. [96] and MRSA in the Netherlands [97]. In fact, in the latter case infection with MRSA was traced back a pig farm, where it was isolated from the pigs and co-workers, and onward transmission to the farm family was documented [97]. Correlation studies and phylogenetic analyses also provide evidence supporting a transmisison of MRSA between animals and human contacts [22, 98, 99]. In Canada, the rate of ceftiofur-resistant *Salmonella* Heidelberg infections was statistically significantly associated with contamination rates on retail chicken [63].

Taken together, the evidence for a link between foodborne or zoonotic bacteria with antimicrobial resistance genes on farms or feedlots and human health risks is undisputable. Exposure to farms, consumption of raw or undercooked foods of animal origin, and living in rural areas have been identified as risk factors for infections with those foodborne pathogens that carry antimicrobial resistance genes [87, 88, 90, 91]. Outbreaks of foodborne illness associated with resistant bacteria have been traced back to the farm of origin [94–96]; identical bacterial strains have been identified in animals and humans in contact with them or the food they produce [97–99]. And in at least some cases, increased intensity of animal contact led to a measurably increased risk of human infection with resistant strains [92, 93]. While the magnitude of the risk may vary across pathogens and circumstance, it is clear that foodborne or zoonotic bacteria on farms or feedlots that carry resistance genes pose a human health risk.

#### Transmission of commensal bacteria from food-producing animals to humans

Most of the research on the emergence of resistance as a result of on-farm use of antimicrobial drugs has been conducted on commensal bacteria, and observations from studies which simultaneously analyzed resistance emergence in foodborne as well as commensal bacteria suggest that resistance may emerge more quickly in the latter. The question of whether the emergence of resistance among commensal or animal pathogens on farms poses a human public health risk is therefore an important one.

As shown in Table 6, a variety of studies have evaluated the link between antimicrobial resistance in animal commensals and human health risks. The available evidence types include observational studies and other study designs such as correspondence of bacterial strains from different sources. Notably, because of the complexity of the events evaluated, study designs are often intricate and may include, for instance, a randomized controlled trial of chicken exposed to antimicrobials, paired with an observational study evaluating the colonization of their human caretakers [100]. While some studies choose prevalence of resistance as study outcome, others also include degree of resistance, expressed for instance as MIC. Farm workers as well as workers in slaughterhouses tend to be the primary population subgroups of concern in these studies, even though consumers handling potentially contaminated carcasses, as well as animals in contact with infected animals have also been considered in some studies [100–106]. Studies also vary in the bacterial population of concern and range from narrowly defined, for instance *E. coli* carrying nalidixic acid resistance [100] to broad, for example anaerobic bacterial populations in general [107]. Conjugation studies are a special study type relevant in this context, which evaluates the transfer rates of bacterial resistance genes between bacterial populations in vivo*,* in the gut of animals or human volunteers [108–112], or in vitro [107].

**Table 6:** Examples of study types evaluating the human health risk posed by antimicrobial resistance genes that emerged in animal pathogens or commensal bacteria

Reference	Study									Key findings relevant to antimicrobial resistance
	Type^a^	subjects	country	time period	bacterial target	relevant study outcomes	sample size	controls	comments	
GRADE level: Controlled trial										
Not available										
GRADE level: observational study										
[100]	CC	poultry workers	Nigeria	published in 1989	*E. coli,* nalidixic acid resistant	colonization of poultry workers with challenge *E. coli* strain	Complex study design: - Birds were experimentally inoculated with challenge strain of *E. coli* (*n* = 36 birds on university farm, and *n* = 16 on commercial farm) - Poultry works in direct contact with challenged birds were sampled for colonization with challenge strain - This was compared to control workers with or without direct contact to control birds, and to samples collected from the exposed workers prior to exposure - birds were sampled for colonization			after birds were challenged, the poultry workers in direct contact with challenged birds were colonized; results were similar on the university and commercial farm
[101]	CC	poultry farmers	U.S.	published in 1978	aerobic bacterial cultures, tetracycline resistance	resistance in bacterial microflora of chicken and contact farmers	Complex study design: - RCT of chicken, *n* = 50 chicken per group, test chicken were exposed to tetracycline in feed, controls were not; emergence of resistance was tested in commensal bacteria - farm family in contact with chicken was tested for emergence of resistance in background microflora - farm family was compared to other farm families in proximity, and to medical students			antimicrobial resistance emerged in exposed chicken and farm contacts
[102]	cohort	poultry farmers	U.S.	published in 1976	intestinal bacterial flora, tetracycline resistance	resistance in bacterial microflora of chicken and contact farmers	*n* = 11 farm members, 24 neighbors	neighbors	birds were exposed to tetracycline in feed, emergence of resistance was traced in birds and contact humans, and compared to neighbors	antimicrobial resistance emerged under exposure on the farm; the prevalence of antimicrobial resistance was higher in bacteria from exposed farm families than in neighbors;
[103]	CS	pig farmers and abattoir workers	Netherlands	published in 1994	*E. coli*	Prevalence of resistance	*n* = 290 pig farmers; *n* = 316 abattoir workers; *n* = 160 urban/suburban residents	urban/suburban residents	fecal *E. coli* from the three human groups were tested for resistance to multiple antimicrobial drugs	Pig farmers had the highest percentage of resistant *E. coli*, and urban/suburban residents had the lowest
[104]	CC	turkey, broiler and layer farmers and slaughterers	Netherlands	published in 2001	*E. coli*	Prevalence and degree of resistance	*n* = 47 turkey farmers and their flocks; *n* = 51 broiler farmers and *n* = 50 broiler flocks; 25 layer farmers and their flocks; *n* = 46 poultry slaughterers	comparison across human (and corresponding animal) populations; ciprofloxacin-resistant isolates were further subtyped; meat samples were collected immediately after slaughter	humans and birds sampled; antibiotic use on farms recorded	Prevalence of resistance significantly higher in turkey and broiler samples than laying hens (which correlated with antibiotic use); prevalence of resistance was higher in turkey and broiler farmers and slaughterers than in laying hen farmers; isolates from farmers/ slaughterers and birds / meat seemed to match, despite variability across farms
GRADE level: other										
[63]	correlation study	isolates from chicken meat and humans	Canada	2003–2008	*Salmonella* Heidelberg, *E. coli*	Prevalence of ceftiofur-resistance in chicken samples and human cases	Correlation across provinces and time periods	n/a	n/a	Statistically significant correlation between ceftiofur-resistant *Salmonella* Heidelberg on retail chicken and human infections; in Quebec, changing levels of ceftiofur use in hatcheries during study period seemed to impact dynamics from before voluntary withdrawal to reintroduction.
[105]	uncontrolled transmission study	farmers, livestock and environment	U.S.	published in 1990	*E. coli*	Spread of resistant bacteria on farm; emergence of resistance	2 trials with *n* = 2 cattle each; *n* = 4 pigs, *n* = 5 mice per cage; bovine experiments repeated in indoor / outdoor settings one challenged pig exchanged with one unchallenged pig from unchallenged pen	N/A; study monitored contact animals, mice in pens of challenged and non-challenged pig or cow, environment including flies, and human caretakers study also evaluated impact of chlortetracycline use on emergence of resistance	one pig and one cow were inoculated with marked strains of *E. coli* with resistance; contact animals (pigs or cows and mice) were sampled for the *E. coli* strain, as were human caretakers and the environment	Contact animals, mice, flies and caretakers excreted challenge strain of *E. coli*; length of colonization varied, but in several cases exceeded 4 weeks; *E. coli* strain was found in environment and housing system impacted spread; chlortetracycline use led to increased resistance; transfer of resistance plasmid to other bacteria not detected
[106]	uncontrolled transmission study	Consumer handling chicken carcasses	US	published in 1977	*E. coli*	spread of resistant bacteria from contaminated carcass to volunteer handling it	1 trial	N/A		Bacterial strain with antibiotic resistance transferred from chicken carcass to human volunteer handling it
[107]	Conjugation study	Transfer of resistance genes among bacteria of different origin	Norway	Published in 1994	*E. coli* as well as *Vibrio, Aeromonas*	Conjugation frequencies across bacteria and environmental settings	Conjugation was studied in multiple environments (e.g., seawater, hand towel, meat)	N/A		Plasmids were readily transferred from one bacterium to another, across bacterial species and in a number of different environments
[108]	Conjugation study	Transfer of resistance genes between *Enterobacteriaceae*	UK	Published in 2003	*Commensal E. coli, E. coil O157, Salmonella*	Conjugation across bacterial species	Conjugation across a number of bacterial pairs was evaluated under conditions in the gut (i.e., ileum)	N/A		Antibiotic resistance genes were readily transferred from one bacterial strain to another
[109]	Conjugation study	Transfer of resistance genes among *Enterococci* in the mouse gut	France	Published in 2003	*Enterococcus faecium isolates from humans and pigs*	Conjugation across *Enterococci* of different origin	In the gut of mice	N/A	mice were gnotobiotic (i.e. reared under conditions so that colonization with bacteria is fully known)	Vancomycin resistance was readily transferred from porcine to human isolates in the gut of mice; tylosin exposure (through drinking water) favored colonization due to conjugation.
[110]	Conjugation study	Transfer of resistance genes between *Enterococci* strains	Sweden	Published in 2006	*Enterococci faecium of human and animal origin*	Conjugation across *Enterococci* of different origin	In vitro and in the gut of mice	N/A	Mice were germ-free	Vancomycin resistance was readily transferred among *Enterococci* of different origin; conjugation frequency was higher in the mouse gut than in the environment; in most cases resistance disappeared within 3 days but one of the bacterial strains persisted for more than 20 days without antibiotic selection
[111]	Conjugation study	Transfer of resistance genes in the gut of human volunteer	Denmark	Published in 2006	*Enterococcus faecium*	Conjugation between *Enterococcus faecium* of chicken and human origin	*n* = 6 volunteers	n/a	In the gut of human volunteers	Resistance genes were readily transferred from chicken to human isolates in the gut of human volunteers; in one volunteer, additional resistance genes were transferred
[112]	Conjugation study	Transfer of resistance from *Klebsiella* to *E. coli* in the mouse intestine	Denmark	Published in 2008	*Klebsiella* and *E. coli*	Conjugation between *Klebsiella* and *E. coli*	In vitro and in the gut of mice	n/a	Some mice were exposed to antimicrobial treatment	Resistance genes were readily transferred in vitro and in vivo. Antimicrobial treatment selected for resistant strains, which rapidly disappeared in the guts of mice not exposed to antibiotics.
[113]	uncontrolled transmission study	Calves and humans in contact with them	US	published in 1978	*E. coli*	Spread of resistant bacteria from calves to human contacts, and impact of tetracycline exposure on risk	1 trial repeated 3 times	N/A	calves were inoculated with rare *E. coli* strain carrying resistance genes	Bacteria were transferred from calves to human contacts; no statistically significant impact of tetracycline exposure on transmission risk; exposure of calves to tetracycline did not result in statistically significant differences in resistance levels of bacteria in human volunteers
[114]	correlation study	Cattle and human contacts	Norway	1996	*E. coli*	Presence of drug-resistant *E. coli* in animals and human contacts	*n* = 13 cattle; *n* = 3 family members; *n* = 1 veterinarian (sampling of humans repeated after 1 year, and samples from 4 other veterinarians added)	N/A	study of concurrence of *E. coli* strains	Multi-drug resistant *E. coli* were found in the animals and human contacts.
[115]	correlation study	Poultry farmers and their birds	Norway	1998	*Enterococcus faecium (VRE)*	Genetic relatedness of human and animal isolates	*n* = 5 farmers and *n* = 7 broiler chicken	N/A	study of concurrence of *E. coli* strains	On one of the farms, human and animal bacteria were genetically closely related; genetically unrelated strains shared related vancomycin resistance genes, suggesting HGT
[116]	correlation study	farm families and their animals (i.e., cattle or swine)	US	Published in 1975	*E. coli*	Genetic relatedness of human and animal isolates	*n* = 14 farm families	N/A	Farm families in Missouri; impact of factors such as animal contact	Frequency of animal contact was not significantly correlated with genetic relatedness of human and animal isolates; consumption of home-raised beef appears to be associated with concordance between human and animal isolates
[118]	Conjugation study	Transfer of resistance genes from *E.coli* from pig to those from human gut	Denmark	2007–2008	*E. coli*	Conjugation between resistance genes among *E.coli* of pig and human origin	*n* = 9 human volunteers;	n/a	donor strain of *E.coli* from Danish pig; recipient strain *E.coli* of human origin	Transfer of resistance genes between *E.coli* bacteria in human intestine
[119]	Conjugation study	Transfer of mobile genetic elements associated with MRSA	UK	Published in 2014	*Staphylococcus aureus* bacterial populations	Frequency of horizontal gene transfer of pig and human origins	Gnotobiotic piglets	n/a	Co-colonization with human and pig associated variants of MRSA	High frequency of horizontal gene transfer; transfer of mobile genetic elements from pig to human within 4 h of co-colonization
[120]	Phylogenetic study	Presence of tetQ resistance gene in different bacteria	US	Published in 1994	*Bacteroides*	Sequence comparison for tetQ resistance gene	n/a	n/a	Presence of identical or closely related resistance genes across distantly related bacteria	Virtually identical resistance genes found in different bacterial species and isolates of different geographic origin

^a^ *CC* case-control study (or case-case study where controls are cases of infection with other strains), *CS* cross-sectional study, *ET* experimental trial (i.e., no randomization and no controls)

A number of observational studies have investigated the effect of antimicrobial exposures on farms on resistance in commensal bacteria isolated from farm animals and human contacts. In one cohort study in the U.S., for instance, when broiler chicken were exposed to tetracycline in the feed, the emergence of tetracycline resistance was traced in the intestinal bacteria isolated from the birds, and in commensal bacteria isolated from 11 members of the farms where the exposed birds were housed [102]. Resistance to tetracycline in the intestinal bacteria from farm members in contact with the exposed birds was compared to that of 24 neighbors. Antimicrobial resistance emerged under exposure on the farm and the prevalence of antimicrobial resistance was higher in bacteria from exposed farm families than in neighbors. In another study of poultry farmers in the U.S. a randomized controlled trial design was combined with a case-control study design [101]. In the randomized, controlled part of the study, groups of 50 chicken were exposed to tetracycline in feed while controls did not receive any antimicrobial drugs. The emergence of resistance in the commensal bacteria on the chicken was tracked. At the same time, the commensal bacteria of farm families in contact with the chicken was tested for the emergence of resistance, and this was compared to the commensal bacteria from other farm families in the proximity, and to commensal bacteria from medical students. The study clearly demonstrated that antimicrobial resistance emerged in exposed chicken and the farm family contacts. Similarly, in a study of 47 turkey, 51 broiler and 25 layer farmers and their flocks as well as 46 poultry slaughterers in the Netherlands, the prevalence and degree of resistance in *E. coli* populations was analyzed and antibiotic use on the farms was recorded [104]. The prevalence of resistance was significantly higher in *E. coli* samples from turkey and broiler flocks than in those from laying hens, which was correlated with antibiotic use practices. The prevalence of resistance was also higher in bacteria from turkey and broiler farmers and slaughterers than in laying hen farmers. Individual isolates from farmers or slaughterers and birds or their meat seemed to match, despite some variability across farms. In another cross-sectional study, the prevalence of resistance in *E. coli* isolates from 290 pig farmers and 316 abattoir workers in the Netherlands was compared to that of 160 urban or suburban residents [103]. Fecal *E. coli* from the three human groups were tested for resistance to multiple antimicrobial drugs. The highest percentage of resistant *E. coli* was found in pig farmers, while urban/suburban residents had the lowest prevalence. In another very complex study design, the colonization of poultry workers with a specifically marked *E. coli* strain was evaluated [100]. Birds were experimentally inoculated with a challenge strain of *E. coli* (36 birds on a university farm, and 16 on a commercial farm). Poultry workers in direct contact with the challenged birds were sampled for colonization with the challenge strain. The results were compared to control workers with or without direct contact to control birds, and to samples collected from the exposed workers prior to exposure. In addition, the birds were sampled for colonization. The study clearly showed that after birds were challenged, the poultry workers in direct contact with the challenged birds were colonized; results were similar on the university and commercial farm.

Evidence for a transmission risk from farm animals to humans is also provided by other study types. For instance, a number of studies lack proper negative controls and apply complex study designs but, despite these limitations, provide evidence in support of a transmission risk. For instance, one study performed in the U.S. evaluated the spread of resistant *E. coli* to livestock, farmers and the environment [105]. In separate experiments, one pig and one cow were experimentally inoculated with a specific *E. coli* strain that carried a particular resistance to make it easier to detect. Animals in contact with the inoculated animals (pigs or cows and mice) were sampled for the *E. coli* strain, as were human caretakers and the environment. Contact animals, mice, flies and caretakers were found to excrete the challenge strain of *E. coli*. The length of colonization varied, but in several cases exceeded 4 weeks. The *E. coli* strain was also found in the environment and housing system and this finding impacted the dynamics of spread. Exposure to chlortetracycline use in the experiments led to increased resistance, even though transfer of resistance plasmid to other bacteria was not detected. The number of animals in this experiment was low (Table 6) and only the cattle experiment was repeated once, with one ‘replicate’ in indoor and one in outdoor settings. Nonetheless, the experiments provide evidence that transmission of resistant bacteria from livestock to human contacts can and does occur. In another uncontrolled transmission study, also conducted in the U.S., the spread of resistant *E. coli* from calves to human contacts was evaluated, and the impact of tetracycline exposure on the risk was assessed [113]. In this case, the experiment was repeated three times. Calves were inoculated with a rare *E. coli* strain carrying resistance genes and human volunteers in contact with the calves were sampled for carriage of the challenge strain. The study provided evidence that challenge bacteria were transferred from calves to human contacts. However, no statistically significant impact of tetracycline exposure on the transmission risk was detected. The exposure of calves to tetracycline did not result in a statistically significant difference in resistance levels of bacteria in the human volunteers. In yet another uncontrolled study, the transmission risk associated with consumer handling of contaminated carcasses was assessed [106]. An *E. coli* strain with an antimicrobial resistance marker was used to inoculate a chicken carcass, and the volunteer handling the carcass was evaluated for carriage of the marked strain. The study clearly showed that the *E. coli* strain was transferred from the chicken carcass to the volunteer handling it. Even though this study also lacked controls and replicates it does provide evidence that bacteria can be transferred to consumers through the handling of contaminated meat or poultry products.

Results from correlation studies, which compare bacterial populations in food producing animals and human contacts, also support a transmission risk even though the evidence is less-well controlled and less rigorous. In one study in Norway, for instance, 13 cattle, and 3 family members in contact with the cattle as well as the veterinarian, were evaluated for the presence of antimicrobial-resistant *E. coli*, and the sampling of human volunteers was repeated after one year, at which point samples from 4 other veterinarians were added [114]. The study found concurrent strains of multi-drug resistant *E. coli* in the animals and human contacts. In another Norwegian study, poultry farmers and their birds were sampled for vancomycin-resistant *Enterococcus faecium* and the genetic relatedness among the bacterial isolates was assessed [115]. On one of the farms, human and animal bacteria were genetically closely related. Notably, even genetically unrelated strains shared related vancomycin resistance genes, suggesting HGT. In another study in the U.S., 14 farm families in Missouri and their livestock (i.e., cattle or swine) were sampled for *E.coli* and the genetic relatedness among the human and animal isolates was assessed [116]. The frequency of animal contact was recorded and its potential impact on the genetic relatedness among the isolates was assessed. Notably, the frequency of animal contact was not significantly associated with the genetic relatedness among human and animal isolates. However, consumption of home-raised beef appeared to be associated with concordance between human and animal isolates.

Taken together, the data clearly support the notion that antimicrobial-resistant commensal bacteria that emerged in food-producing animals can be and are transferred to humans. This may occur through direct contact with the animals, or indirectly, for instance through handling of contaminated carcasses. Notably, in cases of transmission through direct contact directionality may not always be clear and in some instances bacteria may also be transmitted from humans to the animals they are in contact with. Nonetheless, the fact that commensal bacteria can be transferred to humans from food-producing animals is undoubtable.

#### Risk of resistance gene transfer from food-animal associated commensal bacteria to human pathogens

Resistance that emerged in commensal bacteria on farms or feedlots poses a human health risk if it can be transferred to human pathogens. This transfer may occur in the human gut, in the environment, or in the food-producing animals. The ability of human commensals to transfer their resistance genes to human pathogens has been extensively studied and is clearly possible, even though certain transfers are clearly more likely than others (see [117] for a review). Therefore this study focuses on evidence for a transfer of resistance genes between bacteria of human and animal origin regardless of the human-pathogenic potential of the human-associated recipient strain.

A number of in-vivo and in-vitro studies have investigated the ability of commensal bacteria of animal and human origin to transfer their resistance genes. In one study of six human volunteers, for instance, the transfer of resistance genes between *Enterococcus faecium* strains of chicken and human origin was investigated [108]. Resistance genes were readily transferred from chicken to human isolates in the gut of the human volunteers. Notably, in one volunteer, additional resistance genes were also transferred. In another study, transfer of vancomycin resistance between *Enterococcus faecium* isolates of human and swine origin was investigated in the gut of gnotobiotic mice (i.e., mice reared under conditions so that colonization with bacteria is fully known) [106]. Vancomycin resistance was readily transferred from porcine to human isolates in the gut of the mice. Notably, tylosin exposure through drinking water favored colonization with strains that had undergone conjugation. Similar results were observed in another study, which evaluated the transfer of vancomycin resistance between *Enterococci faecium* strains of human and animal origin in the environment and in the gut of germ-free mice [107]. Vancomycin resistance was readily transferred among *Enterococci* of different origin. Notably, the frequency of conjugation was higher in the mouse gut than in the environment. In most cases, resistance disappeared within 3 days but one of the bacterial strains persisted for more than 20 days in the absence of antimicrobial exposure.

Exchange of resistance genes between isolates of animal and human origin has also been demonstrated for other bacteria [118]. In one study, for instance, the transfer of resistance genes from a multi-drug resistant *Klebsiella* strain isolated from a human pneumonia patient to a mouse-adapted *E. coli* strain was investigated in the mouse intestine and under in-vitro conditions [112]. Some of the mice were exposed to antimicrobial drugs during the study. Resistance genes were readily transferred in vitro and in vivo. Antimicrobial exposure selected for resistant strains, which rapidly disappeared in the guts of mice not exposed to antibiotics. Even though transfer in this case occurred from human to animal –associated bacteria it clearly demonstrates that the exchange of genetic material is possible. In another study, the transfer of resistance genes between commensal *E. coli* and two pathogens (*E. coli* O157 and *Salmonella)*, all of porcine origin, was investigated in an in-vitro model of the porcine gut [108]. Antimicrobial resistance genes were readily transferred between the bacteria and in one example persisted throughout the duration of the study. In yet another study, the transfer of resistance genes among *E.coli*, *Vibrio* and *Aeromonas* was investigated in a number of environments (e.g., meat, seawater, hand towel) [107]. Plasmids were readily transferred from one bacterium to another, across bacterial species and in a number of different environments. Similar findings have also been reported for MRSA. In one study, for instance, gnotobiotic piglets were co-colonized with human- and pig-associated variants of MRSA ST 398 and the bacterial populations were followed for 16 days and analyzed by whole-genome sequencing [119]. Transfer of mobile genetic elements from the pig to the human isolates was detected as early as 4 h after co-colonization. Plasmids and bacteriophages were extensively and repeatedly transferred among the isolates, indicating a very high frequency of horizontal gene transfer. Interestingly, bacterial populations differed considerably across pigs, implying limited transfer among the pigs in this study. In another study, nine human volunteers ingested an *E. coli* strain of human origin that carried resistance to rifampicin but was susceptible to sulfonamides. This was followed three hours later by ingestion of another *E. coli* strain, of pig origin and carrying sulfonamide resistance. Stool samples from the volunteers were collected 24 h prior to the challenge, and on days 1 to 7 as well as 14 and 35 after. Conjugation was detected on day 2 in one of the volunteers, mediated through the exchange of a plasmid containing the sulfonamide resistance gene *sul2*, and co-transfer of ampicillin resistance was also demonstrated. Notably, however, the *sul2* resistance gene was identical to one carried by commensal bacteria present in the gut of the volunteer before the experiment but differed from that carried by the pig strain, demonstrating the complexity of these types of investigations, in particular if the resident bacterial populations are not fully known or controlled. A number of other studies have also investigated the transfer of various resistance genes among a diverse group of bacteria in the guts of various animals and other environments and found ample evidence that resistance genes are readily transferred (see [117] for a review). In addition, phylogenetic studies provide further support for horizontal gene transfer of resistance by identifying virtually identical resistance genes in distantly related bacterial species from varying geographic regions [120].

Taken together, there is considerable evidence that resistant animal-associated commensals can and do, at least transiently, colonize humans that are in contact with the animals or, in some cases, their food and that these commensals can transfer resistance genes to human-associated commensals and to human pathogens. Notably, antimicrobial exposure seems to favor the selection and maintenance of conjugates with newly-acquired antimicrobial resistance genes, even though this selection pressure does not seem to be necessary in all cases. There is also strong evidence that the same resistance genes are present in bacteria from animals and humans. What has so far remained less clear is how frequently this transfer occurs under real-world conditions, what other factors such as management practices or antibiotic exposures favor or limit this transfer, exactly when, where and how the transfers occur, and what fraction of resistance genes present in populations of human pathogens originated in commensals on farms.

### Excess morbidity and mortality caused by antimicrobial resistance traits that emerged on farms

To fully evaluate the impact of antimicrobial resistance as a public health burden, it is important to quantify the excess morbidity and mortality caused by the fact that a certain pathogen is resistant to antimicrobial drugs. This section focuses on antimicrobial resistant zoonotic and foodborne pathogens. However, the origin of resistance genes is complex and it is not clear that the resistance genes carried by these foodborne or zoonotic pathogens necessarily emerged in animal agriculture rather than other settings such as human healthcare. Regardless of the origin of the resistance genes, the studies are relevant to the question of whether infections with antimicrobial-resistant bacteria pose an increased public health risk.

For ethical reasons, RCTs comparing health outcomes associated with resistant as compared to susceptible human pathogens are not feasible. However, observational studies as well as other study types such as outbreak analyses are available. Observational studies typically compare outcomes such as hospitalizations or deaths among patients infected with resistant or susceptible strains.

A number of observational studies have compared health outcomes for patients infected with susceptible versus resistant strains of foodborne or zoonotic pathogens such as *Salmonella*, *Campylobacter* or *Staphylococcus aureus* (Table 7). One study in the U.S., for instance, compared the frequency of hospitalization and bloodstream infections in patients with resistant and susceptible *Salmonella* strains [121]. Resistant isolates were significantly more likely to be associated with bloodstream infections than susceptible isolates. For example, for serotype Typhimurium, 3% of patients infected with susceptible isolates had bloodstream infections, compared to 6% of patients infected with resistant strains. Patients infected with resistant isolates also were more likely to be hospitalized because of bloodstream infection than those infected with susceptible isolates. Among the hospitalized patients, those infected with resistant isolates had longer stays than those infected with susceptible isolates. Similar results were obtained in another U.S. based study of 875 patients with gastro-intestinal and bloodstream infections due to *Salmonella* [122]. Bloodstream infections and hospitalizations were significantly more likely among patients with resistant compared to susceptible *Salmonella* isolates. Results from yet another U.S. based study of patients infected with *Salmonella* further support this finding [123]. Patients with resistant infections were significantly more likely to be hospitalized than those with susceptible infections. Notably, recent antimicrobial treatment as well as age and race were significant predictors of resistance.

**Table 7:** Examples of study types evaluating the health impacts associated with human infections due to resistant as compared to susceptible bacterial strains

Reference	Study									Key findings relevant to antimicrobial resistance
	Type^a^	subjects	country	time period	bacterial target	relevant study outcomes	sample size	controls	comments	
GRADE level: Controlled trial										
n/a										
GRADE level: observational trial										
[121]	Cohort	Patients with *Salmonella* infections	US	1996–2001	*Salmonella*	Frequency of hospitalization and bloodstream infection	*n* = 1415 patients (FoodNet) and *n* = 7370 isolates (NARMS)	Patients infected with susceptible *Salmonella* strains	Two analyses were performed, one of *Salmonella* isolates collected through NARMS, and one of patients with NARMS *Salmonella* isolates and corresponding FoodNet interviews	Resistant isolates were more likely associated with bloodstream infections than susceptible isolates (for example, for serotype Typhimurium, 3% of patients infected with susceptible isolates had bloodstream infections, compared to 6% of patients infected with resistant strains); patients infected with resistant isolates were more likely to be hospitalized because of bloodstream infection than those infected with susceptible isolates; among hospitalized patients, those infected with resistant isolates had longer stays than those infected with susceptible isolates;
[122]	Cohort	Patients with *Salmonella* infections (bloodstream and GI infection)	US	2006–2008	*Salmonella*	Clinical outcomes	*n* = 875 patients	Patients infected with susceptible *Salmonella* strains		Bloodstream infections and hospitalizations more likely among patients with resistant than susceptible isolates
[123]	Cohort	Patients infected with *Salmonella*	US	1989–1990	*Salmonella*	Infection and hospitalization		Patients infected with susceptible *Salmonella* strains	Results were compared to similar study conducted in 1979–1980	Patients with resistant infections were more likely to be hospitalized than those with susceptible infections; recent antimicrobial treatment as well as age and race were significant predictors of resistance
[124]	Cohort	Patients with *Salmonella* infections	Canada	1999–2000	*Salmonella Typhimurium*	Risk of hospitalization	*n* = 440 patients	Patients infected with susceptible *Salmonella* strains		Hospitalization was more likely in patients infected with resistant than susceptible strains; an estimated 57–72% of hospitalizations were attributable to resistance patterns
[125]	Cohort	Patients infected with *Salmonella* Typhimurium	Denmark	1995–1999	*Salmonella Typhimurium*	Death rates	*N* = 2047	10 random controls (matched by age, sex and county of residence); comparison between susceptible and resistant infections	Subjects were followed for up to 2 years after entering the study	Patients with susceptible *Salmonella* Typhimurium infection were 2.3 times more likely to die during the study period than the general population; patients with most resistant strains were 4.8 times (95% CI: 2.2–10.2) more likely; patients infected with quinolone-resistant isolates were 10.3 times more likely to die than the general population.
[126]	Cohort	Patients with *Campylobacter* infections	Denmark		*Campylobacter (quinolone and erythromycin-resistant)*	Risk of invasive illness and death within up to 90 days of sample receipt	*n* = 3471 patients	Patients infected with susceptible *Campylobacter* isolates		Patients infected with quinolone-resistant *Campylobacter* strains had a 6-fold increased risk of invasive illness or death within 30 days of sample collection compared with patients infected with susceptible isolates; infection with erythromycin-resistant *Campylobacter* strains was associated with >5-fold higher risk within 90 days compared to infections with susceptible strains
[127]	Meta-analysis of cohort studies	Studies of patients with bloodstream infection due to *Staph. aureus*	multiple countries	1980–2000	*Staphylococcus aureus, MRSA*	Mortality rates due to infections with methicillin susceptible and resistant *Staphylococcus aureus*	*n* = 3963 patients in *n* = 31 studies	Patients infected with methicillin susceptible strain	Results pooled with random effects model	Increased mortality associated with MRSA infections compared to methicillin –susceptible strains (OR: 1.93; 95% CI – 1.52 – 2.42)
GRADE level: other										
[128]	Outbreak analysis	Outbreaks of patients infected with *Salmonella*	US	1984–2002	*Salmonella*	Hospitalization due to infection with susceptible or resistant *Salmonella* strains	*n* = 23,206 patients in *n* = 32 outbreaks	Outbreaks with susceptible *Salmonella* strains		The median proportion hospitalized for each type of outbreak caused by resistant strains was 26%, 2.5 times higher than the median proportion hospitalized for outbreaks caused by pan-susceptible strains

^a^ *CC* case-control study (or case-case study where controls are cases of infection with other strains), *CS* cross-sectional study

Another observational study, conducted in Canada to compare the risk of hospitalization in response to infection with resistant *Salmonella* Typhimurium strains found similar results [124]. Hospitalization was significantly more likely in patients infected with resistant compared to susceptible strains. In fact, an estimated 57–72% of hospitalizations were attributable to the resistance patterns. A Danish study of patients infected with *Salmonella* Typhimurium infections that occurred between 1995 and 1999 evaluated death rates in patients infected with *Salmonella* Typhimurium strains carrying various antimicrobial resistance genes [125]. For every patient enrolled in the study, 10 random controls were found, matched by age, sex and county of residence. Subjects were followed for up to 2 years. Patients with susceptible *Salmonella* Typhimurium infection were 2.3 times (95% CI 1.5 to 3.5) more likely to die during the study period than the general population. By comparison, patients infected with the most resistant strains were 4.8 times (95% CI: 2.2–10.2) more likely, and patients infected with quinolone-resistant isolates were 10.3 times (95% CI 2.8-37.8) more likely to die than the general population.

Similar results have also been obtained for other bacteria. One Danish study of *Campylobacter* infections, for instance, compared the risk of invasive illness and death within up to 90 days of sample collection in 3471 patients infected with quinolone and erythromycin resistant or susceptible strains [126]. Patients infected with quinolone-resistant *Campylobacter* strains had a 6-fold increased risk of invasive illness or death within 30 days of sample collection compared with patients infected with susceptible isolates. Similarly, infection with erythromycin-resistant *Campylobacter* strains was associated with more than a 5-fold higher risk within 90 days compared to infections with susceptible strains. A meta-analysis of cohort studies focused on patients with bloodstream infections due to *Staphylococcus aureus* in multiple countries found similar results [127]. Data from a total of 3963 patients from 31 individual studies were analyzed, comparing mortality rates due to infections with methicillin susceptible and resistant strains. A significantly increased mortality rate was associated with MRSA infections compared to methicillin –susceptible strains (OR: 1.93; 95% CI – 1.52 – 2.42). Data from outbreaks further support worse health outcomes for infections with resistant compared to susceptible strains (see for instance [128]), even though potential confounding effects such as virulence differences among strains or differences in exposed populations have to be considered.

Taken together, there is unequivocal evidence, from epidemiological studies, meta-analyses, and the analysis of outbreaks, that infections with antimicrobial resistant bacteria tend to be associated with worse public health outcomes than infections with susceptible strains. Notably, the impact appears to differ by pathogen as well as resistance involved [121, 126]. The underlying reasons may not always be clear, but can include delayed treatment onset, the need to choose less-desirable treatment options, and, at least in some cases, increased virulence of antimicrobial drug resistant strains because of co-selection for resistance and virulence genes. Regardless of what drives the increased public health cost, infections with antimicrobial resistant bacteria are a public health threat that needs to be addressed.

## Conclusions

The review clearly demonstrates that there is ample scientific support, based on observational studies, a variety of other relevant scientific evidence, and, where ethically feasible, also RCTs to support each step in the causal pathway from antimicrobial use on farms to the public health burden caused by antimicrobial resistant infections in humans. These studies reach across the major food-producing species and a variety of bacterial species including *Salmonella*, *Campylobacter*, *E. coli* and *Enterococci* even though there seem to be some important differences among the bacterial species and data are not readily available for all bacteria, species and antimicrobial drugs of interest. Yet, taken together, the data support what the scientific community, national governments, and international organizations such as the World Health Organization, the Food and Agricultural Organization of the United Nations, and the World Organization for the Health of Animals (OIE) have long recognized: antimicrobial use on farms clearly contributes to the emergence of resistance and poses a human public health risk.

Because the molecular and evolutionary determinants of antimicrobial resistance are complex, resistance does not strictly follow a simple epidemiological model of causation; rather, a more sophisticated model typically used for chronic diseases applies. For example, antimicrobial use may not in all cases lead to the immediate emergence of resistance, and its discontinuation may not lead to an immediate drop in resistance. The antimicrobial drug and treatment regimen as well as external factors such as the feed type used on a given operation can have significant impacts on resistance, and rates of resistance emergence may be markedly different across bacterial species or even strains.

Bacterial resistance genes can be readily shared across bacteria, and some may be pre-existing or persist in environmental reservoirs such as soils. Tracing their origin back across bacterial backgrounds is challenging, even though technological advances such as whole-genome sequencing and more advanced phylogenetic and population genetic models make their trace-back increasingly feasible. Currently, it may be impossible to exactly quantify the contribution of animal agriculture to the overall burden of resistance, but this goal is becoming increasingly feasible.

Importantly, even if some data gaps remain to be filled, there is no doubt that antimicrobial use on farms or feedlots contributes to the problem of antimicrobial resistance. It is therefore important to take action now to ensure antimicrobial drugs are used judiciously, and only when needed to ensure animal health and well-being. Almost 50 years have passed since the Swann report called for the discontinuation of growth promotion uses for medically important antibiotics, and the practice has just now been phased out in the U.S. Many of the studies reviewed here are not new – in fact, many well-designed, compelling studies were published more than 20 or 30 years ago. Time is running out on curtailing antimicrobial resistance. Antimicrobial use on farms contributes to the burden of antimicrobial resistance. Science has shown it, many times over, and stakeholders around the world have come to accept it. It is time to move the public debate away from the problem to its potential solutions.

## Abbreviations

GRADE: Grades of recommendations assessment, development and evaluation
HGT: Horizontal gene transfer
JETACAR: Joint expert technical advisory committee on antibiotic resistance
MIC: Minimum inhibitory concentration
MRSA: Methicillin resistant *Staphylococcus aureus*
OIE: World Organization for Animal Health
RCT: Randomized controlled trial
VRE: Vancomycin resistant *Enterococci*
